# Robust loading and delivery of functional small RNAs *via* protein N-myristoylation-induced small extracellular vesicles

**DOI:** 10.1016/j.bioactmat.2026.04.012

**Published:** 2026-04-13

**Authors:** Huijie Wang, Xiaozhe Zhang, Yunjun Liang, Zekai Zeng, Lianru Bi, Yiying Yang, Jiajie Pan, Gang Dai, Guifu Wu, Wendong Fan

**Affiliations:** a Department of Cardiology, The Eighth Affiliated Hospital of Sun Yat-sen University, Shenzhen, Guangdong, People's Republic of China; b Department of Cardiology, The First Affiliated Hospital of Sun Yat-sen University, Guangzhou, Guangdong, People's Republic of China; c NHC Key Laboratory of Assisted Circulation and Vascular Diseases (Sun Yat-sen University), People's Republic of China; d Guangdong Innovative Engineering and Technology Research Center for Assisted Circulation, Shenzhen, Guangdong, People's Republic of China; e MOE Key Laboratory of Laser Life Science & Guangdong Provincial Key Laboratory of Life Science, College of Biophotonics, School of Optoelectronic Science and Engineering, South China Normal University, Guangzhou, 510631, Guangdong, People's Republic of China

**Keywords:** Extracellular vesicles, RNA therapeutics, N-Myristoylation, siRNA delivery, miRNA delivery

## Abstract

Small extracellular vesicles (sEVs) are naturally secreted nanovesicles that mediate intercellular communication by transporting biomolecules such as proteins and nucleic acids. Their inherent biocompatibility makes them promising platforms for RNA therapeutics; however, efficient encapsulation of small RNAs remains challenging. To address this, we developed the Protein N-Myristoylation-induced sEVs Loading (PMEVL) system. PMEVL employs a genetic construct encoding an N-Myristoylation peptide and, optionally, a small-RNA expression cassette in its 3′-untranslated region, enabling N-myristoylation-dependent, efficient, and specific RNA loading into sEVs. Mechanistically, PMEVL enhances sEVs biogenesis by activating ERK1/2 and inhibiting AMPK, while promoting RNA loading through recruitment of ANXA2 and key ESCRT components ALIX and TSG101. This system achieved highly efficient encapsulation of diverse functional RNAs, including exogenous/endogenous small RNAs (miRNAs, siRNAs) and messenger RNAs (e.g., GFP, mCherry), as well as co-loading of multiple siRNAs with proteins of interest. To demonstrate therapeutic potential, PMEVL-mediated delivery of Pcsk9 siRNA suppressed hepatic Pcsk9 expression *in vitro* and *in vivo*. In C57BL/6 mice, this treatment restored hepatic low-density lipoprotein receptor (LDLR) expression and significantly reduced serum levels of low-density lipoprotein cholesterol (LDL-C) and total cholesterol, without systemic toxicity. Furthermore, systematic screening of 181 peptides representing the N-terminal 15-18 residues of human N-myristoylated proteins identified candidates that substantially enhanced PMEVL loading efficiency. Collectively, PMEVL represents a versatile, efficient, and modular platform for loading RNA therapeutics into sEVs, with demonstrated co-loading capability for proteins *in vitro*.

## Highlights

• PMEVL enables N‑myristoylation‑dependent, efficient loading of diverse RNAs (miRNA, siRNA, mRNA) into sEVs.
• PMEVL boosts sEV biogenesis via ERK1/2 activation & AMPK inhibition, and drives RNA loading through ANXA2, ALIX and TSG101.
• PMEVL supports co‑loading of multiple siRNAs and proteins into sEVs.
• PMEVL‑delivered Pcsk9 siRNA safely restores hepatic LDLR and lowers serum LDL‑C and total cholesterol in mice.
• Screening of 181 human N‑myristoylated peptides identified candidates that optimize the PMEVL platform.

## Graphical abstract

The Protein N-Myristoylation-induced sEVs loading (PMEVL) system employs engineered N-myristoylated-peptides to enhance small extracellular vesicles (sEVs) production and actively package functional exogenous or endogenous small RNAs (miRNAs and siRNAs) into sEVs. PMEVL functions through a dual mechanism: it promotes sEVs biogenesis through ERK1/2 activation and AMPK-autophagy inhibition, while facilitating RNAs loading by recruiting the RNA-binding protein ANXA2 and ESCRT components (ALIX/TSG101). Therapeutically, PMEVL-mediated delivery of Pcsk9 siRNA effectively silenced hepatic Pcsk9 in mice, restored low-density lipoprotein receptor (LDLR) expression, and lowered serum cholesterol without systemic toxicity.

**Figure:**
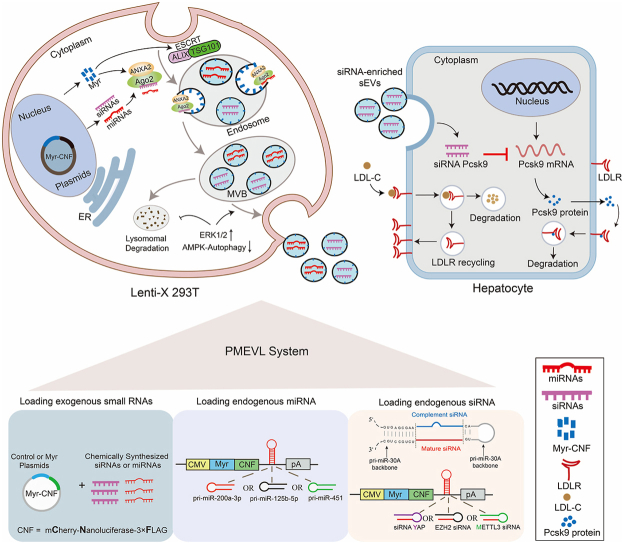

## 1 Introduction

MicroRNAs (miRNAs) and small interfering RNAs (siRNAs) are classes of small non-coding RNAs (19-25 nucleotides) that suppress gene expression post-transcriptionally *via* RNA interference (RNAi), offering significant therapeutic potential [1]. Despite this promise, unmodified RNA molecules face inherent challenges, including poor stability, high immunogenicity, and limited cellular uptake [2]. Delivery systems such as viral vectors, synthetic lipid nanoparticles (LNPs), and polymers have improved RNA stability and delivery efficiency by enhancing nuclease resistance and promoting cellular internalization [2,3]. However, clinical translation of RNA therapeutics is still hindered by limitations of traditional delivery systems, including unwanted immune responses, high cytotoxicity, inefficient delivery, and poor biocompatibility [2,4].

Engineered small extracellular vesicles (sEVs) emerge as promising alternatives for RNA delivery. These naturally secreted lipid nanoparticles (30 to 200 nm in diameter) mediate intercellular communication by delivering biomolecules such as proteins, lipids, and nucleic acids [[5], [6], [7]]. As endogenous carriers, sEVs exhibit favorable properties including low toxicity, low immunogenicity, and the ability to cross biological barriers, making them attractive platforms for sustained *in vivo* drug delivery in clinical situations requiring repeated administration [8,9]. sEVs represent versatile delivery platforms engineered from diverse biological sources for therapeutic cargo [10,11]. Nevertheless, robust and reproducible approaches for efficiently loading therapeutic small RNAs into sEVs remain limited.

Strategies for loading therapeutic cargo into sEVs are broadly classified into exogenous (loading after sEVs isolation) and endogenous (loading during sEVs biogenesis) approaches [2]. Exogenous methods, such as sonication, electroporation, or freeze-thaw cycles, demonstrate utility but suffer from cumbersome protocols, low loading efficiency, and potential damage to sEVs integrity [2,12]. In contrast, endogenous strategies—by genetically engineering sEVs-producing cells to package therapeutic RNAs during sEVs biogenesis—represent a more promising direction [13]. Previous studies reported that RNA incorporation into sEVs can be achieved by designing fusion proteins combining sEVs-sorting domains with RNA-binding proteins (RBPs) that bind target RNAs [14,15]. This approach is effective for loading mRNA, as RBP-interacting elements can be placed in non-coding regions (e.g., the 3′UTR) without affecting translation. However, it is technically complex and poorly suited for small RNAs, since appending such elements to their short sequences may impair their native activity. Although specific sequence motifs facilitate miRNA sorting into sEVs [16,17], most therapeutic miRNAs and siRNAs lack such motifs. Alternative strategies, such as incorporating therapeutic siRNAs into the precursor RNA backbone of miR-451 (pre-miR-451) for sEVs loading [18], show promise but are not applicable to miRNA delivery and require optimization for enhanced efficiency.

Protein N-myristoylation (Myr) is a pivotal lipid modification involving the covalent attachment of myristate to the N-terminal glycine of target proteins, which mediates membrane attachment and protein-protein interactions [19]. Previous studies, including our own, have demonstrated that Myr-peptides derived from the N-termini of myristoylated proteins (e.g., CHMP6, SRC) dramatically enhance the encapsulation of large proteins such as CRISPR/Cas9 into sEVs [[20], [21], [22]]. However, whether such Myr-peptides can efficiently co-load therapeutic small RNAs, including miRNAs or siRNAs, remains unexplored. Moreover, the molecular mechanisms underlying their ability to boost sEVs production and cargo encapsulation are still unclear.

To address these questions, we developed the Protein N-Myristoylation-induced sEVs Loading (PMEVL) platform to achieve efficient, robust, and reproducible encapsulation of exogenous or endogenous small RNAs into sEVs. We demonstrate that PMEVL-engineered sEVs deliver functional small RNA cargos, which potently suppress target gene expression in recipient cells both *in vitro* and *in vivo*. Mechanistically, PMEVL functions through two complementary pathways: it enhances sEVs biogenesis by activating the ERK1/2 cascade while suppressing the AMPK-autophagy axis, and it facilitates RNA loading by recruiting the RNA-binding protein ANXA2 and the endosomal sorting complex required for transport (ESCRT) components ALIX/TSG101. Furthermore, to maximize cargo-loading capacity, we systematically screened 181 peptides derived from the N-termini of human N-myristoylated proteins and identified several candidates that significantly enhance PMEVL efficiency. This study presents a novel, modular, and efficient strategy for sEVs-based RNA delivery, thereby advancing the translational potential of RNA therapeutics.

## 2 Results

### 2.1 PMEVL platform facilitates loading of exogenous chemically synthesized siRNAs and miRNAs into sEVs

**Fig. 1:**
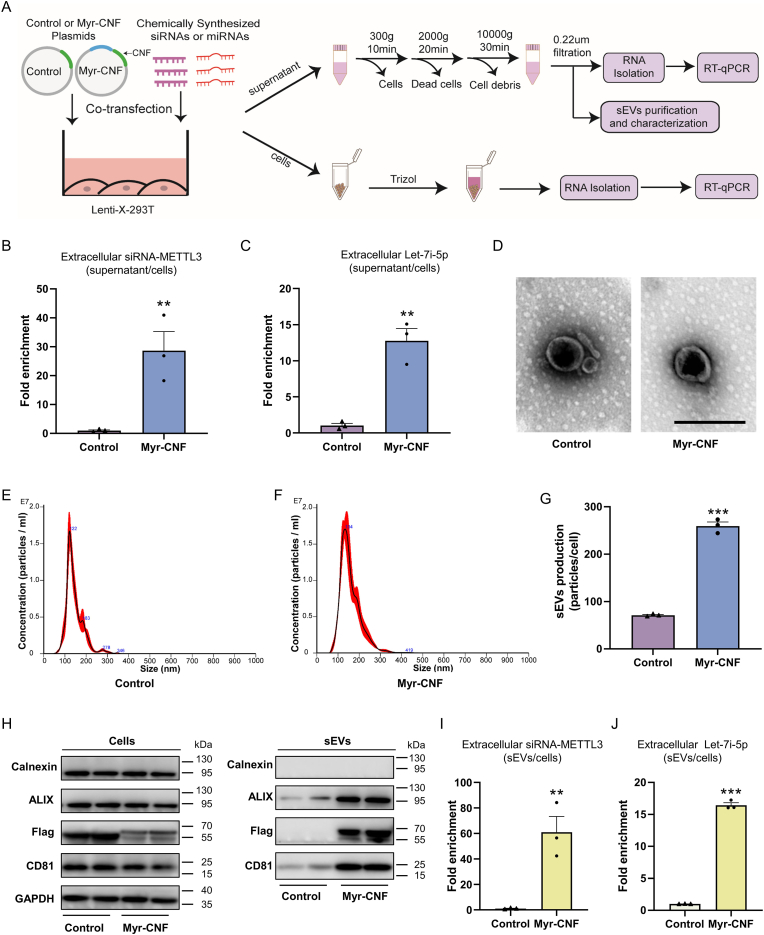
**PMEVL platform enables efficient loading of exogenous chemically synthesized small RNAs into sEVs. A.** Schematic workflow for loading exogenous chemically synthesized siRNA or miRNA into sEVs using the PMEVL platform and subsequent analyses. CNF: Reporter protein (mCherry-NanoLuc-3 × FLAG) for visualization and quantification. Myr-CNF: CNF fused to the C-terminus of an Myr-peptide derived from CHMP6. **B and C.** Fold enrichment of siRNA-METTL3 (B) and let-7i-5p miRNA (C) in cell culture supernatant, calculated as supernatants RNA level/U6-normalized intracellular RNA level in Myr-CNF-transfected cells relative to the corresponding ratio in control cells; n = 3. **D.** Representative transmission electron microscopy images of sEVs isolated from control or Myr-CNF-transfected cells. Scale bar: 200 nm. **E-G.** Size distribution (E and F) and particle (G) of sEVs from control or Myr-CNF-transfected cells, determined by nanoparticle tracking analysis (NTA); n = 3. **H.** Western blot analysis of FLAG-tagged CNF (control)/Myr-CNF, sEVs markers (CD81, ALIX) and endoplasmic reticulum marker calnexin in cell lysates (left) and isolated sEVs (right). **I and J.** Fold enrichment of siRNA-METTL3 (I) and let-7i-5p miRNA (J) in isolated sEVs, calculated as sEVs RNA level/U6-normalized intracellular RNA level in Myr-CNF-transfected cells relative to the corresponding ratio in control cells; n = 3. Data are presented as mean ± SEM. Statistical significance was determined using two-sided unpaired *t-test* (B, C, G, I, and J). ∗*P* < 0.05, ∗∗*P* < 0.01, ∗∗∗*P* < 0.001 versus control.

To investigate the PMEVL strategy, we selected the representative Myr-peptide Myr(CHMP6) [20] and examined its capacity for loading exogenous, chemically synthesized small RNAs, including siRNA-METTL3 (targeting methyltransferase-like 3) and let-7i-5p miRNA. A recombinant reporter protein (designated CNF-tag, comprising mCherry, nanoluciferase (NLUC), and a 3 × FLAG tag) was fused to the C-terminus of Myr(CHMP6) to generate Myr-CNF for visualization and quantitation. We co-transfected exogenous small RNAs with either Myr-CNF or CNF control plasmid into lenti-X 293T cells, and quantified cargo RNA levels in cell lysates, supernatants, and isolated sEVs *via* RT-qPCR (Fig. 1A). Loading efficiency was assessed using fold enrichment, defined as the ratio of extracellular RNA levels (supernatants or sEVs) to U6-normalized intracellular levels. We hypothesized that PMEVL-mediated enhancement of small-RNA cargo loading into sEVs would increase RNA levels in sEVs-derived supernatants from Myr-CNF-transfected cells. Consistent with this hypothesis, Myr-CNF transfection enhanced cargo enrichment in supernatants by ∼28-fold for siRNA-METTL3 (Fig. 1B and S1A) and ∼13-fold for let-7i-5p (Fig. 1C and S1B), demonstrating efficient extracellular RNA enrichment.

To confirm sEVs-associated loading, we isolated sEVs from supernatants *via* ultracentrifugation and characterized them using transmission electron microscopy, nanoparticle tracking analysis, and western blotting (Fig. S1C). sEVs from both groups exhibited typical cup-shaped morphology, with size distributions peaking at 122 nm (control) and 134 nm (Myr-CNF) (Fig. 1D–F). The Myr-CNF group showed increased sEVs yield (Fig. 1G) and elevated FLAG-tagged Myr-CNF expression in isolated sEVs (confirming enhanced protein loading capacity *via* the Myr-peptide), along with enriched sEVs markers CD81 and ALIX and no detectable endoplasmic reticulum marker calnexin (Fig. 1H). These results collectively validate both high-purity sEVs isolation and augmented sEVs production mediated by the Myr-peptide. Notably, Myr-CNF transfection enhanced cargo enrichment in sEVs by ∼60-fold for siRNA-METTL3 (Fig. 1I and S1D) and ∼15-fold for let-7i-5p (Fig. 1J and S1E), demonstrating that PMEVL efficiently loads exogenous chemically synthesized small RNAs into sEVs.

### 2.2 PMEVL platform enhances loading of endogenously expressed miRNAs into sEVs

**Fig. 2:**
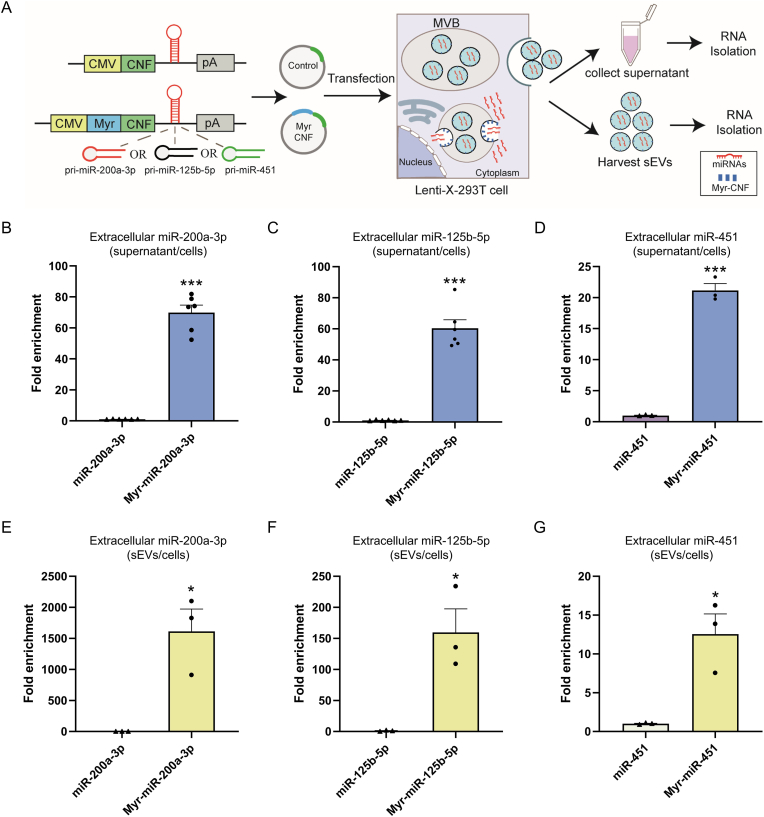
**The PMEVL platform enhances the loading of endogenously expressed miRNAs into sEVs. A.** Schematic of the PMEVL platform for endogenous miRNA loading into sEVs. **B-D.** Fold enrichment of miR-200a-3p (B), miR-125b-5p (C), and miR-451 (D) in cell culture supernatant, calculated as supernatants miRNA level/U6-normalized intracellular miRNA level in Myr-CNF-transfected cells relative to the corresponding ratio in control cells; n = 3-6. **E-G.** Fold enrichment of miR-200a-3p (E), miR-125b-5p (F), and miR-451 (G) in isolated sEVs, calculated as sEVs miRNA level/U6-normalized intracellular miRNA level in Myr-CNF-transfected cells relative to the corresponding ratio in control cells (set as 1); n = 3. Data are presented as mean ± SEM. Statistical significance was determined using a two-sided unpaired *t-test* (B-G). ∗*P* < 0.05, ∗∗*P* < 0.01, ∗∗∗*P* < 0.001 versus control.

To evaluate whether the PMEVL approach facilitates encapsulation of endogenously expressed miRNAs into sEVs, we engineered a co-expression construct containing a cytomegalovirus (CMV)-driven expression unit for Myr-CNF or CNF control, plus a miRNA expression cassette in the 3′ untranslated region (UTR) of the respective transcript to generate cargo miRNAs (miR-200a-3p, miR-125b-5p, or miR-451; Fig. 2A). These miRNAs were selected for their inherent propensity for sEVs loading [[23], [24], [25]]. Post-transfection, cargo miRNA levels in cell lysates, supernatants, and sEVs isolated from control or Myr-CNF-expressing cells were quantified using RT-qPCR. As anticipated, Myr-CNF co-expression significantly enriched cargo miRNAs in supernatants relative to control: ∼69-fold for miR-200a-3p (Fig. 2B and S2A), ∼60-fold for miR-125b-5p (Fig. 2C and S2B), and ∼21-fold for miR-451 (Fig. 2D and S2C). sEVs analysis further confirmed significant enrichment: ∼1616-fold for miR-200a-3p (Fig. 2E and S2D), ∼162-fold for miR-125b-5p (Fig. 2F and S2E), and ∼12-fold for miR-451 (Fig. 2G and S2F), validating efficient endogenous miRNA loading into sEVs.

To assess broader applicability, we evaluated PMEVL-mediated loading of longer RNA cargoes (GFP and mCherry mRNA), which yielded ∼278-fold enrichment for GFP mRNA and ∼16-fold enrichment for mCherry mRNA in sEVs (Fig. S3). Collectively, these findings demonstrate that PMEVL enables loading of diverse RNA cargoes into sEVs.

### 2.3 Protein N-myristoylation enables robust and specific loading and delivery of functional miRNAs

**Fig. 3:**
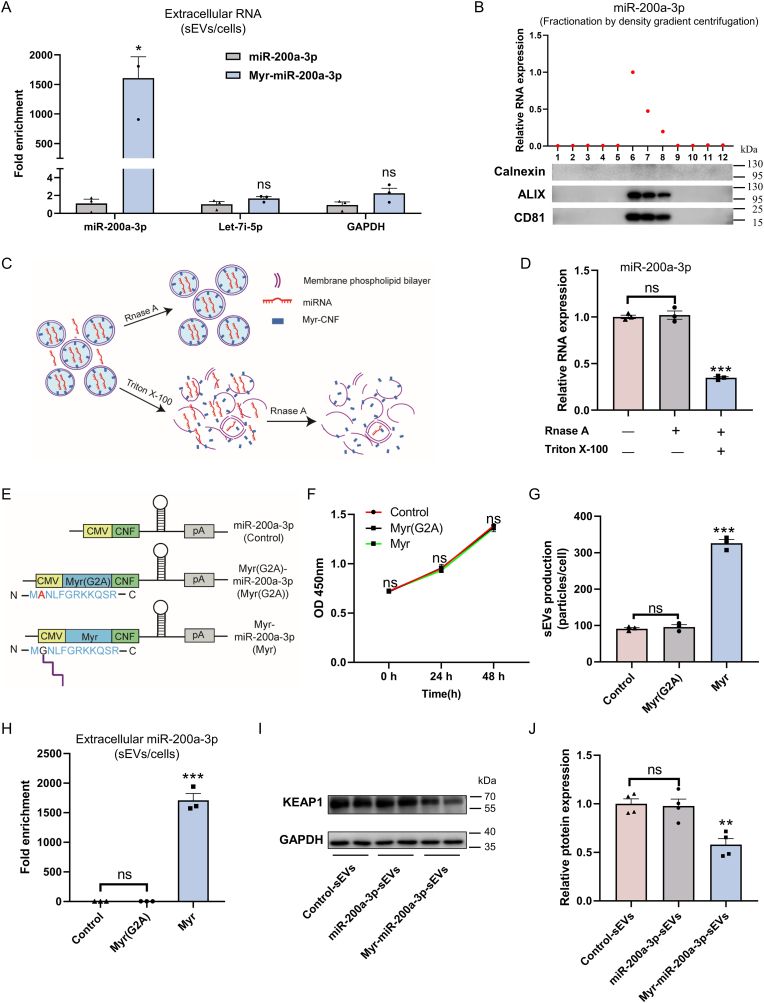
**Protein N-myristoylation enables robust and specific loading and delivery of functional miRNAs. A.** Fold enrichment of indicated RNAs in sEVs isolated from Myr-CNF and miR-200a-3p co-expressing cells (Myr-miR-200a-3p), relative to control cells expressing miR-200a-3p alone. Highly abundant cellular RNAs (let-7i-5p and GAPDH mRNA) served as non-cargo controls; n = 3. **B.** Co-fractionation of miR-200a-3p with sEVs markers shown by iodixanol density gradient; Top: miR-200a-3p distribution (red dots) by RT-qPCR. Bottom: Western blot for sEVs markers (CD81, ALIX) and endoplasmic reticulum marker calnexin. **C.** RNase protection assay workflow. **D.** Relative miR-200a-3p levels in sEVs after indicated treatments; n = 3. **E.** Construct schematics: Control (miR-200a-3p), myristoylation-deficient mutation (Myr (G2A)), and wild type myristoylation (Myr). **F.** Cell viability assessed by CCK-8 assay at 24 h and 48 h post-transfection shows no significant differences between cells expressing the functional Myr-peptide, Myr (G2A) mutant, or negative control; n = 4. **G.** G2A mutation abolishes Myr-CNF-enhanced sEVs production; n = 3. **H.** G2A mutation eliminates Myr-CNF-mediated miR-200a-3p loading; n = 3. **I.** KEAP1 protein in HUVECs after 48 h treatment with sEVs from: Control, miR-200a-3p-transfected, or Myr-200a-3p-transfected lenti-X 293T cells. **J.** KEAP1 levels normalized to GAPDH; n = 4. Data are presented as mean ± SEM. Statistical significance was determined using a two-sided unpaired *t-test* (A) or one-way *ANOVA* (D, F, G, H, and J). ∗*P* < 0.05, ∗∗*P* < 0.01, ∗∗∗*P* < 0.001 versus control.

Among tested RNA cargos, PMEVL exhibited the highest efficiency in loading miR-200a-3p into sEVs, which was thus selected as a model cargo to investigate N-myristoylation-dependent miRNA loading and delivery. As shown in Fig. 3A and S4A-B, co-expression of Myr-CNF with miR-200a-3p (Myr-miR-200a-3p group) significantly enriched miR-200a-3p in sEVs, while endogenous abundant non-cargo RNAs (let-7i-5p and GAPDH) showed minimal enrichment, confirming cargo specificity. To verify the selective loading of miR-200a-3p into membrane-enclosed sEVs (rather than non-vesicular fractions), we performed high-resolution iodixanol gradient fractionation. As expected, miR-200a-3p co-fractionated with sEVs markers CD81 and ALIX (Fig. 3B), confirming specific incorporation into sEVs. Furthermore, RNase protection assays on purified sEVs (Fig. 3C), showed that RNase A alone did not affect miR-200a-3p levels (Fig. 3D), while Triton X-100 permeabilization prior to RNase treatment caused significant degradation (Fig. 3D), indicating that miR-200a-3p is encapsulated within the sEVs lumen. To confirm the requirement for myristoylation, we generated a glycine-to-alanine mutation at position 2 (G2A mutant) to abolish this modification (Fig. 3E). CCK-8 assay confirmed comparable cell viability in cells expressing the functional Myr-peptide, Myr(G2A) mutant, or negative control, with no statistically significant difference observed (Fig. 3F). Critically, the Myr(G2A) mutation abolished PMEVL's ability to enhance sEVs production (Fig. 3G) and package miR-200a-3p into sEVs (Fig. 3H and S4C-D), demonstrating that N-myristoylation is indispensable for robust and specific miRNA loading by PMEVL.

To evaluate the functional delivery of encapsulated miRNAs to recipient cells, we performed *in vitro* uptake experiments. sEVs isolated from lenti-X 293T cell supernatants were co-cultured with human umbilical vein endothelial cells (HUVECs), selected based on our previous identification of endothelial Kelch-like ECH-associated protein 1 (KEAP1) as a functional target of miR-200a-3p [26]. Confocal microscopy confirmed HUVEC uptake of PKH67-labeled sEVs after 24 h incubation (Fig. S4E). Subsequent co-culture (Fig. S4F) and Western blot analysis (Fig. 3I–J), revealed that PMEVL-engineered sEVs (Myr-miR-200a-3p-sEVs) significantly suppressed KEAP1 expression. Collectively, these findings demonstrate that the PMEVL enables N-myristoylation-dependent, robust, and specific loading and delivery of functional miRNAs.

### 2.4 Evaluation of engineered pri-miRNA backbones for endogenous siRNA generation, loading, and delivery

**Fig. 4:**
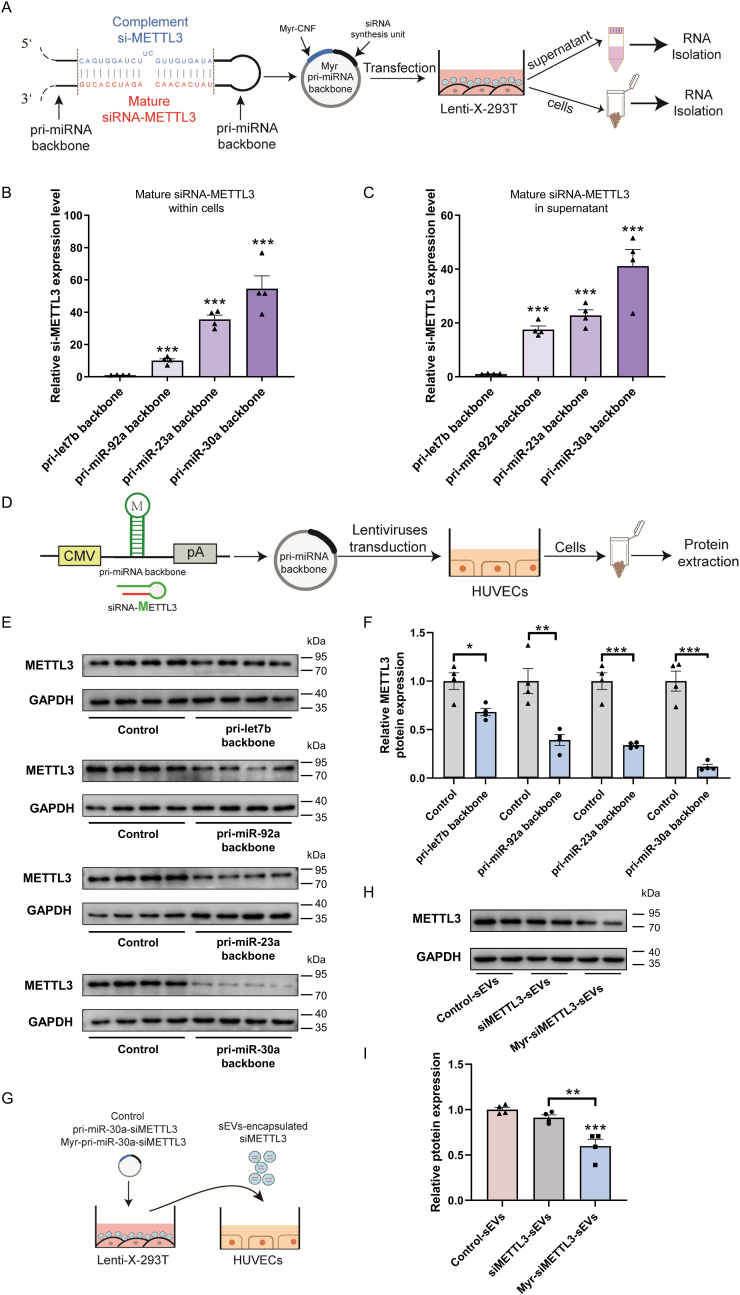
**Evaluation of engineered pri-miRNA backbones for endogenous siRNA generation, loading, and delivery. A.** Schematic of engineered pri-miRNA backbones incorporating METTL3-targeting siRNA. Artificial pri-miRNAs contain a mature siRNA sequence (red) and a complementary strand containing the UC bulge (blue). **B and C.** RT-qPCR quantification of mature siRNA-METTL3 levels in (B) cell lysates and (C) culture supernatants from lenti-X 293T cells transfected with engineered pri-miRNA scaffolds expressing siRNA-METTL3; pri-let7b scaffold served as control; n = 4. **D.** Workflow for Western blot validation of functional METTL3 siRNAs generated from different pri-miRNA backbones. **E and F.** Western blot analysis of METTL3 protein levels in HUVECs transduced with lentiviruses encoding diverse pri-miRNA backbones-based METTL3 siRNAs; n = 4. **G.** Schematic of HUVECs treatment with sEVs derived from lenti-X 293T cells transfected with: Control vectors (control-sEVs), pri-miR-30a-siMETTL3 (siMETTL3-sEVs), or Myr-pri-miR-30A-siMETTL3 (Myr-siMETTL3-sEVs). **H and I.** METTL3 protein levels in sEVs-treated HUVECs by Western blot; n = 4. Data are presented as mean ± SEM. Statistical significance was determined using a two-sided unpaired *t-test* (B, C, and F) and a one-way *ANOVA* (I). ∗*P* < 0.05, ∗∗*P* < 0.01, ∗∗∗*P* < 0.001 versus control.

Given the established capacity of PMEVL for loading endogenously synthesized miRNAs, we next sought to explore its potential for loading and delivery of endogenously expressed siRNAs. Endogenous primary miRNAs (pri-miRNAs) are widely used as scaffolds for intracellular siRNAs synthesis [27]. Artificial miRNAs (AmiRNAs), engineered by inserting siRNA sequences into pri-miRNA backbones, exhibit superior specificity and efficacy over conventional short hairpin RNAs (shRNAs), prompting us to use pri-miRNA scaffolds for siRNA expression. We selected pri-miR-30a as the primary scaffold due to its well-characterized status and the natural enrichment of its mature product (miR-30a) in sEVs [25]. Alternative scaffolds (pri-let7b, pri-miR-92a, and pri-miR-23a) were evaluated based on the enrichment of their mature miRNAs in sEVs [28,29]. Each engineered pri-miRNA scaffold incorporated siRNA-METTL3 (red) on the 3′ strand of the stem structure, with the complementary strand containing a UC bulge (blue) on the 5′ strand (Fig. 4A). Following co-expression of Myr-CNF and pri-miRNA-based siRNA-METTL3 in lenti-X 293T cells, RT-qPCR analysis showed significantly higher siRNA-METTL3 levels in both cell lysates (Fig. 4B) and supernatants (Fig. 4C) when using the pri-miR-30a scaffold. Consistently, siRNA-METTL3 derived from pri-miR-30a exhibited superior silencing efficiency against METTL3 in HUVECs (Fig. 4D–F). Thus, the pri-miR-30a scaffold was selected for subsequent siRNA expression studies.

To evaluate functional delivery, lenti-X 293T cells were transfected with: Myr-pri-miR-30a-siMETTL3 (co-expressing Myr-CNF and siRNA-METTL3), pri-miR-30a-siMETTL3 (co-expressing CNF and siRNA-METTL3), or a control vector (expressing CNF alone). sEVs isolated from these cells were designated Myr-siMETTL3-sEVs, siMETTL3-sEVs, and control-sEVs, respectively (Fig. 4G). Western blot analysis revealed a significant reduction in METTL3 levels in HUVECs co-cultured with Myr-siMETTL3-sEVs compared to controls (Fig. 4H–I). Collectively, these results demonstrate that the pri-miR-30a scaffold enables efficient endogenous generation of functional siRNAs, and that PMEVL effectively packages and delivers these siRNAs *via* sEVs to recipient cells, achieving potent target gene silencing.

### 2.5 The PMEVL system enables simultaneous loading of multiple siRNAs into sEVs

**Fig. 5:**
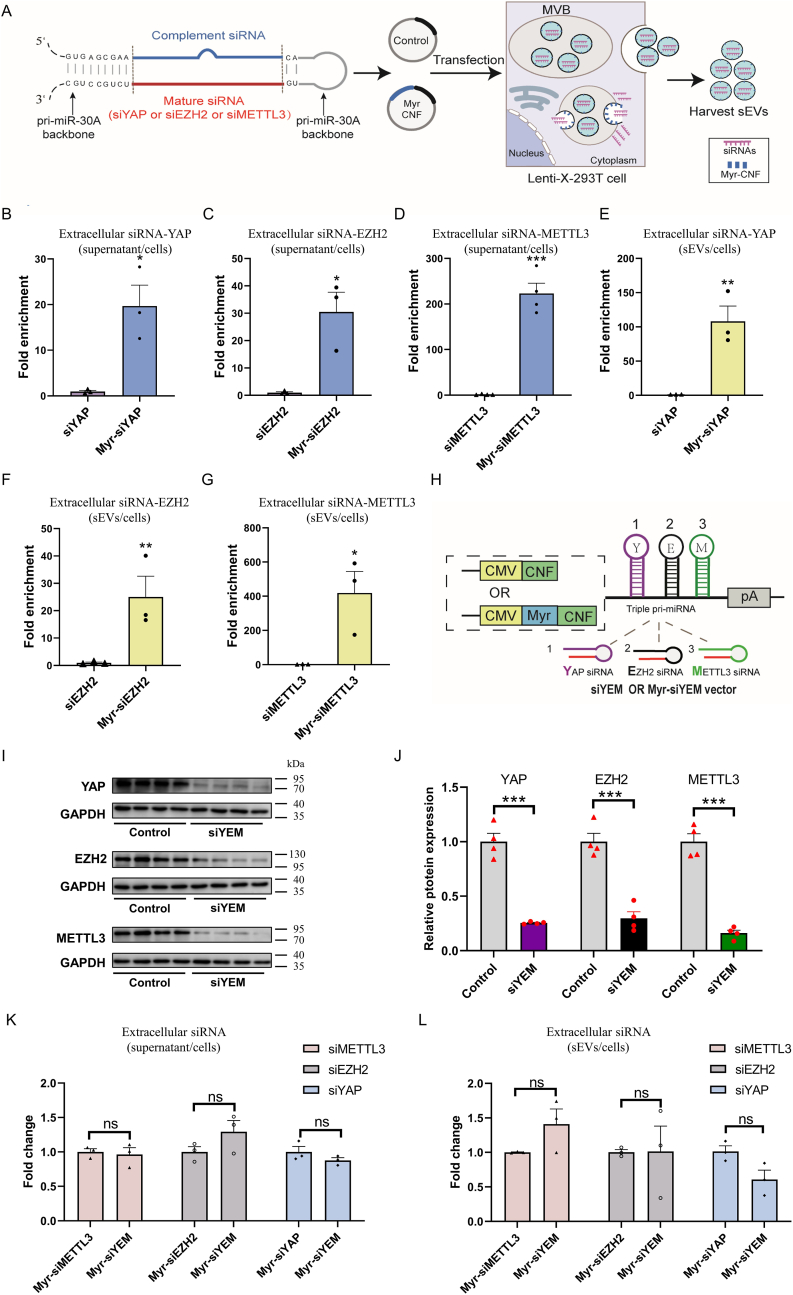
**The PMEVL system enables simultaneous loading of multiple siRNAs into sEVs. A.** Schematic of endogenous siRNA loading using the PMEVL platform. **B-G.** Robust siRNA enrichment in supernatants (B-D: siRNA-YAP (B), siRNA-EZH2 (C), and siRNA-METTL3 (D)) and purified sEVs (E-G: siRNA-YAP (E), siRNA-EZH2 (F), and siRNA-METTL3 (G)). Enrichment was calculated as the siRNA level in supernatant or sEVs divided by U6-normalized intracellular siRNA level in cells transfected with Myr-siRNA constructs (expressing Myr-CNF and cargo siRNA), relative to control cells (expressing CNF with the corresponding siRNA); n = 3. **H.** Tri-cistronic siRNA vector design targeting YAP/EZH2/METTL3 using pri-miR-30a scaffolds. Myr-siYEM expresses Myr-CNF with downstream siYAP/siEZH2/siMETTL3 cassettes; siYEM expresses CNF with equivalent cassettes. **I.** Representative immunoblot of YAP, EZH2, and METTL3 in HUVECs transduced with control or siYEM lentivirus. **J.** Quantification of target protein suppression from I; n = 4. **K and L.** siRNA levels in supernatants (K) and purified sEVs (L) from lenti-X 293T cells expressing indicated constructs; n = 3. Data are presented as mean ± SEM. Statistical significance was determined by a two-sided unpaired *t-test* (B-G and J-L). ∗*P* < 0.05, ∗∗*P* < 0.01, ∗∗∗*P* < 0.001 versus control.

We next tested if PMEVL could load diverse siRNAs expressed *via* the pri-miR-30a scaffold. We incorporated three previously reported siRNAs—targeting yes-associated protein 1 (siYAP) [26], enhancer of zeste homolog 2 (siEZH2) [26], or METTL3 (siMETTL3) [30] into the pri-miR-30a scaffold, which was inserted into the 3′ UTR of either the Myr-CNF or the control CNF (Fig. 5A). RT-qPCR analysis of cell lysates and supernatants showed that Myr-CNF co-expression significantly enriched cargo siRNAs in supernatants relative to control CNF: ∼20-fold for siYAP (Fig. 5B and S5A), ∼30-fold for siEZH2 (Fig. 5C and S5B), and ∼218-fold for siMETTL3 (Fig. 5D and S5C). To validate this further, we isolated sEVs from supernatants and quantified cargo RNA levels. Consistently, Myr-CNF overexpression resulted in significant enrichment of these siRNAs within sEVs: ∼108-fold for siYAP (Fig. 5E and S5D), ∼25-fold for siEZH2 (Fig. 5F and S5E), and ∼419-fold for siMETTL3 (Fig. 5G and S5F). These findings indicate that the PMEVL effectively loads diverse pri-miR-30a-based siRNAs into sEVs.

Since many diseases involve dysregulation of multiple genes, simultaneous modulation of therapeutic targets is critical to enhance efficacy and reduce adverse effects [31]. We thus explored PMEVL's capacity to co-load multiple distinct siRNAs into sEVs. We constructed Myr-siYEM and siYEM vectors, each containing three pri-miR-30a-based siRNA cassettes (siYAP, siEZH2, and siMETTL3) downstream of the Myr-CNF or control CNF, respectively (Fig. 5H). Western blot analysis of HUVECs transduced with control or siYEM lentiviruses confirmed that the pre-miR-30a backbone facilitated simultaneous synthesis of functional siRNAs, as evidenced by marked reductions in YAP, EZH2, and METTL3 protein levels (Fig. 5I–J). Notably, Myr-CNF co-expression enabled release of all three cargo siRNAs into supernatants (Fig. 5K and S6A-B) and their loading into sEVs at levels comparable to single siRNA cargoes (Fig. 5L and S6C-D). Taken together, these results demonstrate that PMEVL effectively enables simultaneous loading of multiple siRNAs into sEVs.

### 2.6 PMEVL enhances sEVs biogenesis and RNA loading by activating ERK1/2, inhibiting AMPK-autophagy, and recruiting ANXA2 and ESCRT machinery

**Fig. 6:**
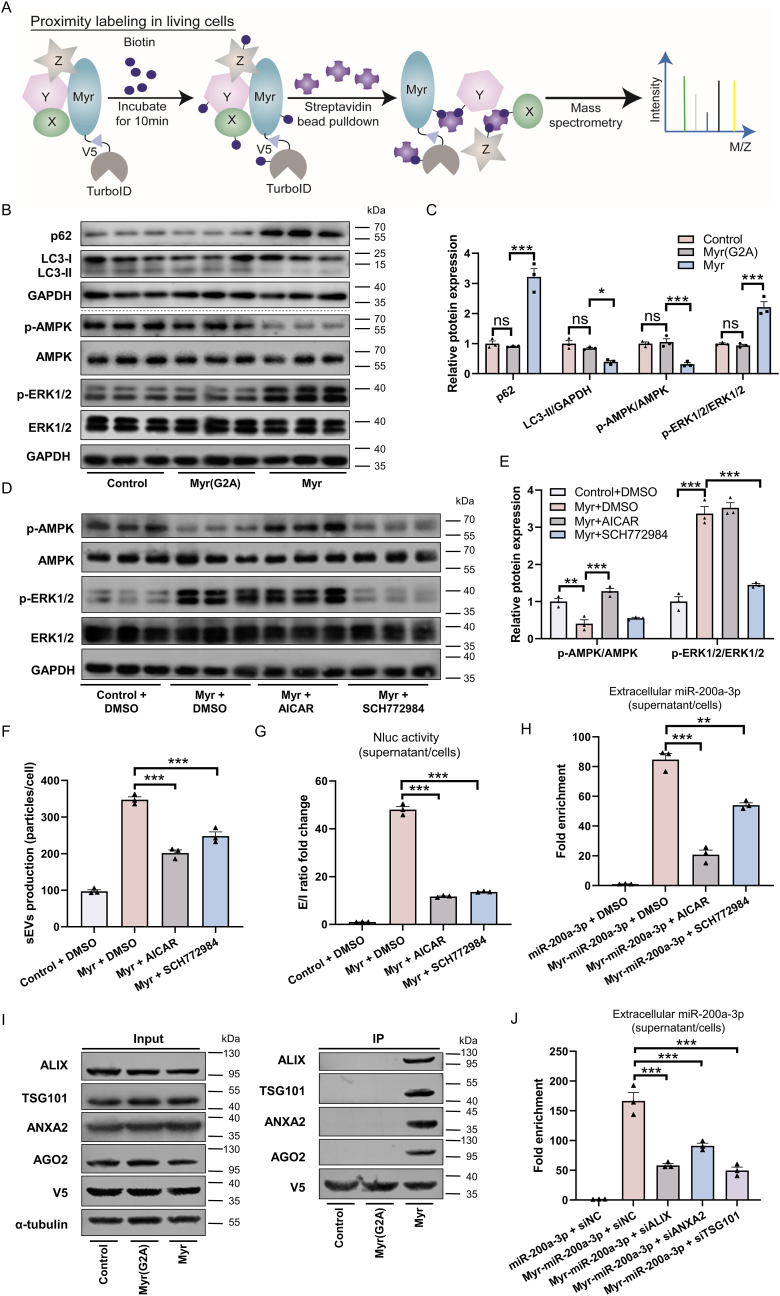
**PMEVL promotes sEVs biogenesis and RNA loading by activating ERK1/2, inhibiting AMPK-autophagy, and recruiting ANXA2/ESCRT machinery. A.** Schematic of the TurboID-mediated proximity labeling workflow coupled with mass spectrometry (MS) analysis. Lenti-X 293T cells expressing Myr-TurboID were treated with 50 μM biotin for 10 min. Cell lysates were incubated with streptavidin magnetic beads to capture biotinylated proximal proteins, which were subsequently identified by MS. **B.** Western blot analysis of p62, LC3-II, phosphorylated AMPK (p-AMPK), AMPK, phosphorylated ERK1/2 (p-ERK1/2), and ERK1/2 expression in lenti-X 293T cells transfected with the indicated constructs: CNF (Control), Myr(G2A)-CNF (Myr(G2A)), or Myr-CNF (Myr); **C.** Quantification of relative protein expression in B; n = 3. **D.** Western blot analysis of p-ERK1/2, ERK1/2, p-AMPK and AMPK in lenti-X 293T cells transfected with CNF (Control) or Myr-CNF (Myr) construct for 24 h, followed by 24 h treatment with: DMSO (vehicle control), AICAR (0.5 mM, AMPK activator), or SCH772984 (0.5 μM, ERK inhibitor); n = 3. **E.** Quantification of relative protein expression in D; n = 3. **F.** sEVs production (particles/cell; NTA quantification) after treatments in D; n = 3. **G.** Protein cargo loading efficiency measured by extracellular/intracellular NanoLuc ratio (E/I) under conditions in D; n = 3. **H.** miR-200a-3p fold enrichment (extracellular miR-200a-3p/U6-normalized intracellular miR-200a-3p) in Lenti-X 293T cells transfected with Myr-miR-200a-3p or miR-200a-3p vectors and treated as in D; n = 3. **I.** Affinity purification analysis based on proximity labeling confirms Myr-peptide interaction with ANXA2, ALIX, TSG101, and AGO2. Lenti-X 293T cells were transfected with indicated constructs: NES-TurboID (Control), Myr(G2A)-TurboID (Myr(G2A)), or Myr-TurboID (Myr). After biotin treatment (50 μM, 10 min), cell lysates were subjected to streptavidin pull-down. Captured proteins were detected by Western blot. V5 signal indicates TurboID fusion protein levels in the whole-cell lysate (Input) and in the affinity-purified samples (pull-down). α-Tubulin served as a loading control for input. **J.** miR-200a-3p fold enrichment (extracellular miR-200a-3p/U6-normalized intracellular miR-200a-3p) in Lenti-X 293T cells transfected with Myr-miR-200a-3p or miR-200a-3p vectors alongside control siRNA (siNC) or siRNA targeting ALIX (siALIX), ANXA2 (siANXA2), or TSG101 (siTSG101); n = 3. Data are presented as mean ± SEM. Statistical significance was determined by one-way *ANOVA* (C) or two-way *ANOVA* (E-H and J). ∗*P* < 0.05, ∗∗*P* < 0.01, ∗∗∗*P* < 0.001 versus control.

To elucidate how the PMEVL system mediates small RNA loading into sEVs, we performed TurboID-based proximity labeling, using the Myr-peptide as bait (Fig. 6A). This identified 1455 proximal proteins (≥2 unique peptides; Fig. S7; Supplemental Excel 1). Bioinformatic enrichment analysis revealed strong associations with key sEVs-related processes, including multivesicular body (MVB) assembly, membrane fission, and exosomal secretion (Fig. S7). Pathway analysis further highlighted the endocytosis, ERK1/2, and AMPK signaling pathway (Figures S8, S9 and S10, respectively), suggesting that the Myr-peptide regulates sEVs biology through these cascades.

Notably, 43 identified proteins are linked to endocytosis (Fig. S7), a process essential for early endosome formation and MVB maturation [32], supporting its role in Myr-peptide-mediated sEVs biogenesis. We also detected five proteins associated with the ERK1/2 cascade and 19 linked to the AMPK pathway (Fig. S7). ERK1/2 signaling is known to promote sEVs biogenesis and secretion by suppressing lysosomal degradation and favoring MVB fusion with the plasma membrane [33]. Conversely, AMPK, a key regulator of autophagy, promotes autophagy progression, which may facilitate MVB-lysosome fusion and reduce sEVs secretion [34]. Consistent with these associations, the Myr-peptide increased phosphorylated ERK1/2 (p-ERK1/2) levels (Fig. 6B–C), indicating activation of the ERK1/2 pathway. Conversely, it downregulated phosphorylated AMPK (p-AMPK) and the autophagy marker LC3-II, while upregulating the autophagy substrate p62 (Fig. 6B–C), indicating suppression of the AMPK pathway and autophagy. Pharmacological inhibition of ERK1/2 (using SCH772984; Fig. 6D–E) or activation of AMPK (using AICAR; Fig. 6D–E) significantly attenuated the Myr-peptide-induced increases in both sEVs production (Fig. 6F) and cargo loading (NLUC protein, Fig. 6G and S12A-B; miR-200a-3p, Fig. 6H and S12C-D)**.** Thus, the Myr-peptide enhances sEVs production by activating ERK1/2 while inhibiting the AMPK-autophagy axis, thereby rediverting MVBs from lysosomal degradation toward secretion.

Among the proximal proteins were 598 RBPs, including the known miRNA delivery mediator annexin A2 (ANXA2) and the ESCRT components ALIX and TSG101 (Fig. S7). Affinity purification analysis based on proximity labeling confirmed interactions between Myr-peptide and ANXA2, ALIX and TSG101; these interactions were abolished by the Myr(G2A) mutation (Fig. 6I). AlphaFold modeling predicted that ANXA2 interacts with argonaute 2 (AGO2), potentially forming ternary complexes with small RNAs where both ANXA2 and the RNAs bind directly to AGO2 (Fig. S11). The high confidence of these models supports the hypothesis that ANXA2 may facilitate small RNA loading into sEVs indirectly *via* AGO2. Consistent with this prediction, proximity labeling-based affinity purification further confirmed interactions among the Myr-peptide, ANXA2 and AGO2 (Fig. 6I). To investigate the functional roles of ANXA2, ALIX, and TSG101 in Myr-mediated miRNA sorting into sEVs, we knocked down these genes using siRNA. Successful downregulation of ANXA2, ALIX, and TSG101 at both mRNA and protein levels was confirmed in siRNA-transfected cells (Fig. S12E–G). Depletion of each protein significantly impaired the ability of the Myr-peptide to promote miR-200a-3p loading into sEVs (Fig. 6J and S12H-I). Critically, ANXA2 silencing selectively impaired miR-200a-3p cargo loading without altering sEVs biogenesis (Fig. S12J). These results indicate that ANXA2 plays a specific and essential role in the miRNA sorting process, while ALIX and TSG101 contribute to the broader sorting of RNA-protein complexes into MVBs during sEVs biogenesis.

Collectively, our findings reveal that PMEVL coordinates sEVs biogenesis and small RNA loading through two mechanisms: promoting sEVs production by activating ERK1/2 and inhibiting AMPK-autophagy to drive sEVs secretion; and facilitating small RNA recruitment by engaging ANXA2 for miRNA loading and the ESCRT machinery (ALIX/TSG101) for RNA-protein complex sorting.

### 2.7 The PMEVL system efficiently delivers Pcsk9 siRNAs to hepatocytes and suppresses Pcsk9 expression *in vitro*

**Fig. 7:**
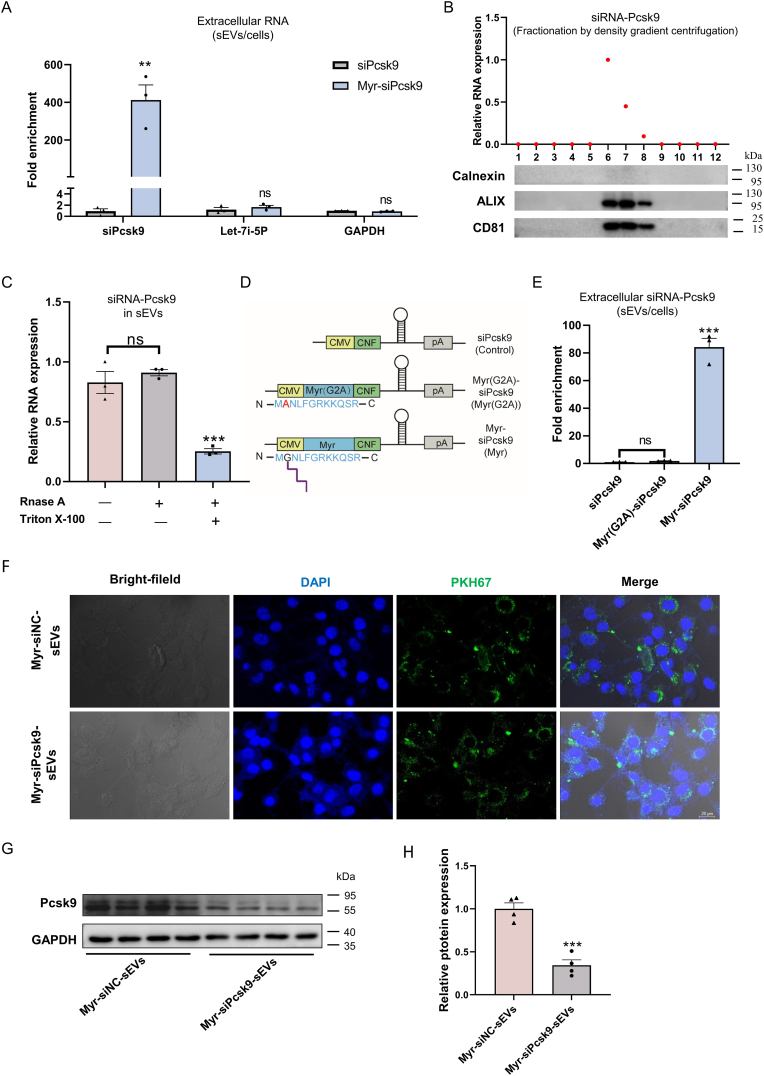
**The PMEVL platform enables specific loading and functional delivery of Pcsk9 siRNA to hepatocytes, suppressing Pcsk9 expression *in vitro*. A.** Robust enrichment of siPcsk9 in sEVs demonstrated by RT-qPCR; fold enrichment calculated as siPcsk9 level in sEVs relative to U6-normalized intracellular levels in Myr-siPcsk9-transfected cells, relative to control cells; n = 3. **B.** Co-fractionation of siPcsk9 with sEVs markers shown by iodixanol density gradient. Top: siPcsk9 distribution (red dots) across fractions measured by RT-qPCR. Bottom: Western blot analysis of CD81, ALIX, and calnexin in gradient fractions. **C.** siPcsk9 is protected within sEVs confirmed by RNase resistance assay: siPcsk9 levels in Myr-CNF-derived sEVs treated with indicated reagents were quantified by RT-qPCR; n = 3. **D.** Schematic of myristoylation site mutations in Myr-CNF. **E.** Myristoylation site mutation abolishes siPcsk9 loading into sEVs; n = 3. **F.** Confocal microscopy of Hepa1-6 cells after 24 h incubation with PKH67-labeled sEVs (green). Nuclei: DAPI (blue). Scale bar: 20 μm. **G and H.** siPcsk9-loaded sEVs suppress Pcsk9 protein expression in Hepa1-6 cells by Western blot; n = 4. Data are presented as mean ± SEM. Statistical significance was determined using a two-sided unpaired *t-tests* (A and H) or one-way *ANOVA* (C and E). ∗*P* < 0.05, ∗∗*P* < 0.01, ∗∗∗*P* < 0.001 versus control.

Proprotein convertase subtilisin/kexin type 9 (Pcsk9), highly expressed and secreted in the liver, accelerates lysosomal degradation of hepatic low-density lipoprotein receptor (LDLR) and is a well-validated therapeutic target for lowering plasma low-density lipoprotein cholesterol (LDL-C) levels [35,36]. We thus explored PMEVL's therapeutic potential by loading and delivering mouse Pcsk9-targeting siRNAs (siPcsk9). As expected, co-expression of Myr-CNF and siPcsk9 enabled robust and specific loading of siPcsk9 into sEVs, without affecting the encapsulation of non-cargo RNAs (endogenously abundant let-7i-5p or GAPDH; Fig. 7A and S13A-B). Furthermore, iodixanol density gradient fractionation demonstrated co-fractionation of siPcsk9 with sEVs markers CD81 and ALIX (Fig. 7B), and RNase protection assay verified siPcsk9 encapsulation within sEVs (Fig. 7C). Mutation of the myristoylation site abrogated Myr-CNF-mediated siPcsk9 loading into sEVs (Fig. 7D–E and S13C-D), confirming N-myristoylation-dependent loading.

Next, we investigated if lenti-X 293T-derived sEVs could be internalized by mouse Hepa1-6 hepatocytes. Confocal microscopy demonstrated uptake of PKH67-labeled sEVs by Hepa1-6 cells after 24 h of co-culture (Fig. 7F). To assess functional delivery of siPcsk9, lenti-X 293T-derived sEVs were co-cultured with Hepa1-6 cells, and Pcsk9 protein levels were measured *via* western blotting. As expected, siPcsk9-loaded sEVs significantly suppressed Pcsk9 expression in Hepa1-6 cells (Fig. 7G–H). In conclusion, PMEVL enables robust and specific loading and delivery of functional siPcsk9 to hepatocytes, effectively inhibiting hepatic Pcsk9 expression *in vitro*.

### 2.8 PMEVL platform enables efficient Pcsk9 siRNA delivery to suppress hepatic Pcsk9, restore LDLR expression, and reduce serum cholesterol in mice

**Fig. 8:**
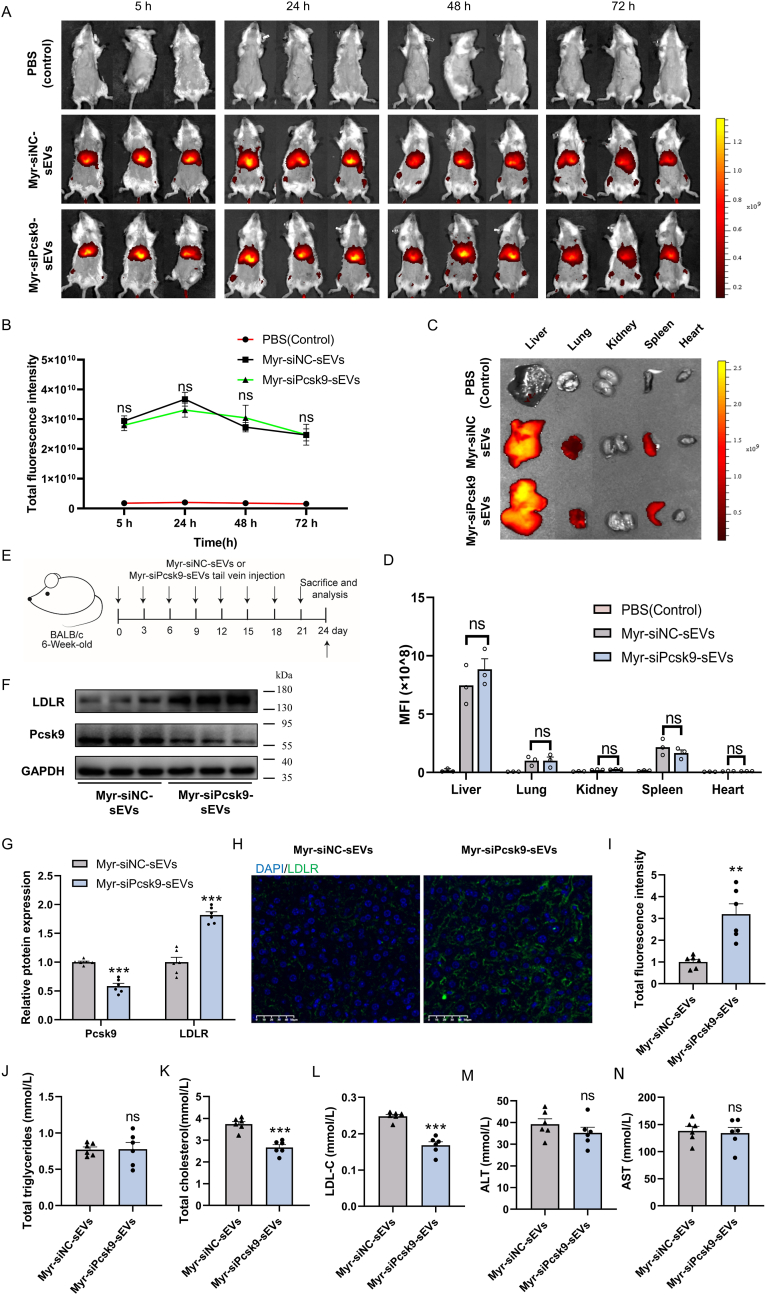
**PMEVL-engineered sEVs deliver Pcsk9 siRNA to hepatocytes, suppressing Pcsk9 expression, restoring LDLR levels, and reducing serum cholesterol *in vivo*. A.***In vivo* fluorescence imaging showing biodistribution of DiR-labeled sEVs after tail vein injection of PBS (Control), Myr-siNC sEVs, or Myr-siPcsk9 sEVs in BALB/c mice. **B.** Quantification of whole-body fluorescence intensity form panel A; n = 3. **C.***Ex vivo* fluorescence images of major organs 72 h post-injection. **D.** Quantification of organ-specific fluorescence intensity from panel C; n = 3. **E.** Experimental design: Myr-siPcsk9 or Myr-siNC sEVs (2.7 ✕ 10^10^ particles per mouse) were administered *via* tail vein injection to 6-week-old C57BL/6 mice every 3 days (8 injections total). Tissues and blood were collected 72 h post-final injection. **F and G.** Western blot analysis of hepatic Pcsk9 and LDLR protein expression; n = 6. **H.** Immunofluorescence of liver sections showing LDLR (green) and DAPI (blue). Scale bar: 50 μm. **I.** Quantification of LDLR fluorescence intensity from panel H; n = 6. **J-N.** Serum analysis of triglycerides (J), total cholesterol (K), low-density lipoprotein cholesterol (LDL-C) (L), alanine aminotransferase (ALT) (M), and aspartate aminotransferase (AST) (N); n = 6. Data are presented as mean ± SEM. Statistical significance was determined using a two-sided unpaired *t-test* (I-N) or a one-way *ANOVA* (B, D and G). ∗*P* < 0.05, ∗∗*P* < 0.01, ∗∗∗*P* < 0.001 versus control.

To evaluate *in vivo* biodistribution of PMEVL-engineered sEVs, DiR-labeled sEVs were intravenously injected into male BALB/c mice. In vivo fluorescence imaging (IVIS Spectrum) showed peak hepatic accumulation at 24 h post-injection, with sustained signal detection up to day 3 (Fig. 8A–B). *Ex vivo* analysis confirmed predominant liver localization, with lower accumulation in the spleen and lungs (Fig. 8C–D), establishing PMEVL-engineered sEVs as effective hepatic delivery vehicles.

To evaluate *in vivo* efficacy, 6-week-old male C57BL/6 mice were intravenously injected with either Pcsk9-siRNA-loaded PMEVL-engineered sEVs (Myr-siPcsk9-sEVs) or control scrambled-siRNA-loaded sEVs (Myr-siNC-sEVs), at a dose of ∼2.7 ✕ 10^10^ particles per mouse, every 3 days for a total of 8 doses (Fig. 8E). Tissues and serum were collected 72 h post-final injection. Western blotting of liver lysates demonstrated significantly reduced Pcsk9 protein and elevated LDLR expression in the Myr-siPcsk9-sEVs group compared to controls (Fig. 8F–G). Immunofluorescence imaging further confirmed increased hepatic LDLR levels (Fig. 8H–I and S14A). Consistent with restored LDLR expression, Myr-siPcsk9-sEVs treatment significantly decreased serum LDL-C and total cholesterol, with no effect on triglyceride levels (Fig. 8J–L). Safety assessments showed no systemic toxicity: serum alanine aminotransferase (ALT) and aspartate aminotransferase (AST) levels remained within normal ranges, and hematoxylin and eosin (H&E) staining of major organs showed no pathological abnormalities (Fig. 8M−N and S14B). These findings demonstrate that PMEVL enables safe, efficient hepatic siRNA delivery; Pcsk9 suppression *via* PMEVL-engineered sEVs restores LDLR expression and reduces circulating cholesterol *in vivo*.

### 2.9 Screening putative human Myr-peptides to optimize PMEVL cargo-loading capacity

The above findings prompted us to investigate if Myr-peptides other than Myr(CHMP6) could serve as more effective sEVs cargo-loading devices. We hypothesized that peptides exhibiting higher accumulation within sEVs post-transfection than Myr(CHMP6) would have superior loading efficiency. To test this, we screened 181 human genome-encoded candidate peptides containing UniProt-annotated N-myristoylation sites. Each candidate was designed as a fusion protein comprising a 15-18 amino acid Myr-peptide and a C-terminal CNF reporter tag for visualization and sensitive quantitation.

**Fig. 9:**
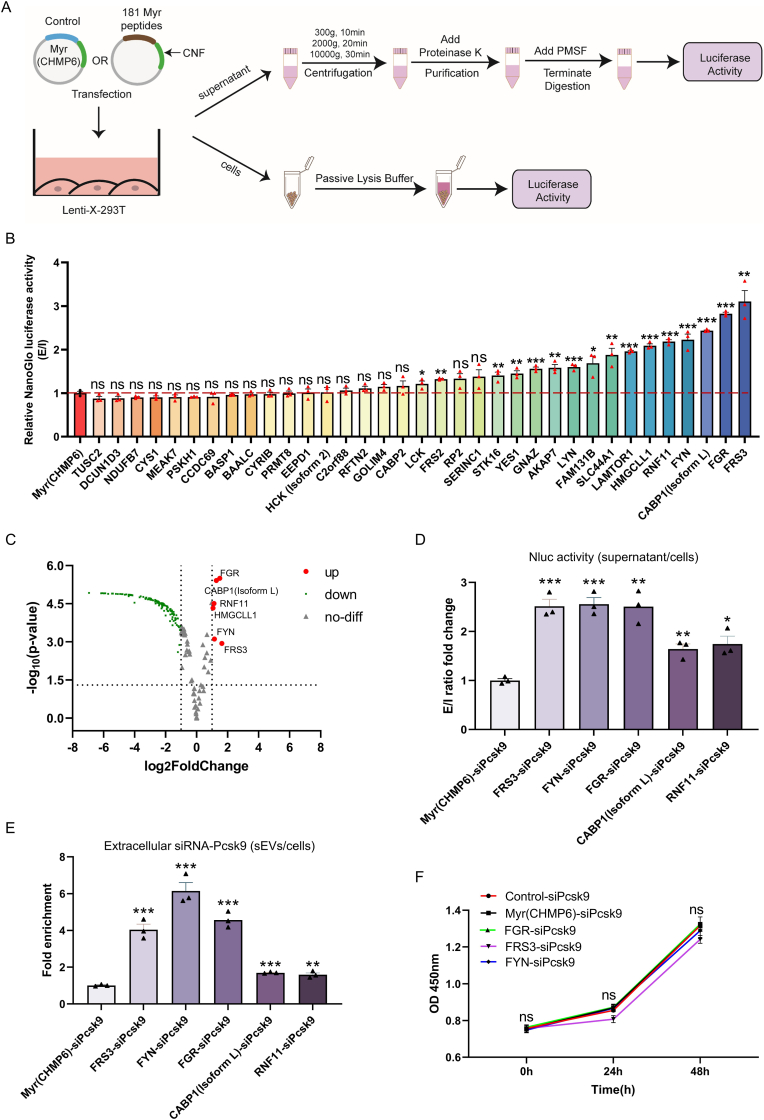
**Systematic screening of human N-myristoylated protein-derived peptides to optimize the cargo-loading capacity of the PMEVL system. A.** Schematic of the Nano-Glo luciferase assay workflow. **B.** Nano-Glo luciferase screening of 181 putative human Myr-peptides for cargo-loading efficiency versus Myr(CHMP6) control. The extracellular-to-intracellular ratio (E/I ratio) was calculated as the luciferase activity in the cell culture supernatant (CS) divided by that in the cell lysate (CL). Red threshold indicates E/I fold-change = 1; n = 3. **C.** Volcano plot distribution of relative luciferase activity (E/I) for screened N-myristoylated proteins. X-axis: log2(fold-change) relative to Myr(CHMP6); Y-axis: significance (-log10(*P*-value)) relative to Myr(CHMP6). **D.** Engineered peptides significantly enhance cargo export: Fold-change in E/I ratio relative to Myr(CHMP6) control measured by Nano-Glo assay; n = 3. **E.** RT-qPCR analysis of Pcsk9 siRNA in sEVs. Fold enrichment was defined as Pcsk9 siRNA level in sEVs divided by U6-normalized intracellular Pcsk9 siRNA level in cells transfected with indicated constructs; n = 3. **F.** Cell viability assessed by CCK-8 assay at 24 h and 48 h post-transfection confirmed unaltered cell viability across all PMEVL variants tested (FGR, FRS3, FYN); n = 4. Data are presented as mean ± SEM. Statistical significance was determined using a two-sided unpaired *t-test* (B, D and E) or a one-way *ANOVA* (F). ∗*P* < 0.05, ∗∗*P* < 0.01, ∗∗∗*P* < 0.001 versus control.

Post-transfection, cargo protein levels in cell lysates and supernatants were quantified *via* luciferase activity (Fig. 9A), with loading efficiency defined as the extracellular-to-intracellular (E/I) ratio (supernatant signal/lysate signal). This screen identified 16 peptides with significantly higher cargo-loading capacity than Myr(CHMP6) and 19 peptides with comparable efficiency (Fig. 9B–C, S15A-B and Supplemental Excel 2). The top five performers (FRS3, FGR, CABP1(isoform L), FYN, and RNF11) were co-expressed with pri-miR-30a-based si-Pcsk9 to evaluate dual protein/RNA cargo-loading capacity (Fig. S15C). As expected, these peptides outperformed Myr(CHMP6) in loading both luciferase protein (Fig. 9D and S15D-E) and si-Pcsk9 RNA (Fig. 9E and S15F-G), as evidenced by higher E/I ratios and sEVs RNA levels. Critically, expression of representative optimized peptides (FRS3, FGR, FYN) showed no statistically significant impact on cell viability (Fig. 9F). Thus, optimization of the Myr-peptide substantially enhances PMEVL efficiency without compromising cell viability.

To further quantify small RNA encapsulation efficiency per sEVs particle with the optimized PMEVL system, we selected miR-200a-3p (the RNA cargo with the highest loading efficiency among those tested) for absolute quantification of miRNA copy numbers per vesicle. A standard curve generated using synthetic miR-200a-3p (Fig. S16A) enabled calculation of absolute miRNA copies per sEVs by normalizing total miRNA copies to sEVs particle counts (determined *via* Nanoparticle Tracking Analysis, NTA). Results revealed significant Myr-peptide-dependent variation in miR-200a-3p loading (Fig. S16B): ∼0.43 copies/sEVs for Myr(CHMP6), ∼0.89 copies/sEVs for FYN, and ∼1.05 copies/sEVs for FGR. Thus, optimized Myr-peptides enhance both loading efficiency and the absolute number of functional RNA cargo delivered per sEVs particle.

## 3 Discussion

Small RNAs hold great clinical potential as therapeutic molecules, but their translation is hindered by the poor stability, low cellular uptake, and high immunogenicity of naked RNA, necessitating effective delivery system [1,2]. sEVs are promising endogenous carriers with superior safety and biocompatibility compared to viral vectors or synthetic carriers [2,3]; however, their natural yield and efficiency in loading therapeutic RNA remain limited. Our PMEVL platform addresses these issues by simultaneously enhancing sEVs production and enabling efficient encapsulation of both exogenous and endogenous functional small RNAs, offering a powerful tool to advance sEVs-based RNA therapeutics.

Most human diseases involve dysregulation of multiple genes or pathways, often requiring simultaneous targeting to improve efficacy and reduce adaptive resistance [31]. The PMEVL platform meets this demand by efficiently loading diverse siRNAs into sEVs and supporting co-loading with proteins of interest; further optimization the Myr-peptide can enhance this cargo-loading efficiency. Notably, despite cargo-dependent variations, the platform achieves comparable loading efficiency for mRNAs (e.g., GFP/mCherry) and small RNAs, highlighting its versatility. These features make PMEVL a reliable system for combinatorial RNA/protein delivery *via* sEVs, with significant potential for treating complex diseases.

To validate the therapeutic potential of the PMEVL system, we targeted Pcsk9, a clinically established, hepatocyte-enriched regulator of LDLR and plasma LDL-C levels [36,37]. Systemically administered PMEVL-sEVs loaded with Pcsk9-targeting siRNA preferentially accumulated in the liver, significantly reducing hepatic Pcsk9 expression and serum LDL-C levels without inducing systemic toxicity, demonstrating promise for liver-targeted therapy. Beyond hepatic applications, the tissue specificity of PMEVL-sEVs could be further extended through surface engineering strategies, such as conjugating targeting ligands or genetically modifying producer cells [38], which may help minimize off-target effects. Furthermore, incorporating chemically modified siRNAs (e.g., those containing phosphorothioate linkages, 2′-O-methyl/fluoro nucleotides, or GalNAc conjugates) [39] into the PMEVL system warrants further investigation. Such modifications could enhance stability and efficacy, addressing key limitations of RNAi therapeutics like transient bioactivity and frequent dosing requirements, thereby potentially reducing cytotoxicity, lowering therapeutic doses, and improving clinical outcomes.

The PMEVL platform likely stimulates sEVs synthesis by modulating endocytosis, ERK1/2 signaling, and the AMPK-autophagy axis, as suggested by the enrichment of Myr-peptide-interacting proteins in these pathways. sEVs biogenesis is linked to endocytosis, which drives early endosomes formation and MVB maturation [32]. MVBs subsequently either fuse with lysosomes for degradation or with the plasma membrane to release sEVs [32]. Activation of ERK1/2 signaling has been reported to promote sEVs secretion by suppressing lysosomal function, thereby shifting MVB fate toward plasma membrane fusion [33]. Conversely, AMPK, a key autophagy regulator, promotes autophagic flux upon activation, which may enhance MVB-lysosome fusion and reduce sEVs secretion [34]. Our data show that the Myr-peptide activates ERK1/2 while suppressing the AMPK-autophagy axis, suggesting that it redirect MVBs from lysosomal degradation toward sEVs release, thereby increasing sEVs yield. Consistent with this model, pharmacological inhibition of ERK1/2 or activation of AMPK significantly reduced PMEVL-induced increases in both sEVs production and cargo loading. Notably, the role of AMPK in sEVs secretion is context-dependent: while its inhibition enhances sEVs release in adipocytes, AMPK activity in other cell types can reduce sEVs secretion *via* mTORC1- and lysosome-dependent regulation of MVB fate [40,41]. Thus, AMPK does not uniformly promote or suppress sEVs secretion, and further studies are needed to clarify its role in specific contexts.

Small RNA loading *via* the PMEVL platform is likely mediated by interactions between the Myr-peptide and specific factors, including ANXA2 and the ESCRT components ALIX and TSG101. ANXA2 is known to facilitate miRNA loading into sEVs [42], while ALIX and TSG101, key regulators of MVB biogenesis, contribute to the sorting of RNA-protein complexes into intraluminal vesicles [43,44]. Our TurboID-based proximity labeling and affinity purification analyses confirmed that the Myr-peptide interacts with ANXA2, ALIX, and TSG101, and that these interactions were abolished by the Myr(G2A) mutation, demonstrating their strict dependence on N-myristoylation. Critically, proximity labeling-based affinity purification further confirmed interactions among the Myr-peptide, ANXA2, and the key RNA-induced silencing complex (RISC) component AGO2. Furthermore, AlphaFold structural modeling predicted that ANXA2 interacts with AGO2 and can form ternary complexes with small RNAs. This combined evidence suggests a mechanism by which the Myr-peptide, *via* ANXA2, may bridge RNA cargo (potentially in complex with AGO2) to the sEVs loading machinery. Collectively, our findings support a dual mechanistic model: the Myr-peptide enhances sEVs biogenesis by activating ERK1/2 signaling and inhibiting the AMPK-autophagy axis to redirect MVBs toward secretion; concurrently, it facilitates RNA loading by engaging ANXA2 for cargo capture and ALIX/TSG101 for vesicular sorting.

Notably, direct comparisons between sEVs platforms can be challenging due to differences in cargo, sEVs sources, and methodologies. However, we benchmarked PMEVL against the widely used pre-miR-451 scaffold [18], which typically achieves ∼0.01 mature miR-451 copies per sEV as measured in natural sEVs. PMEVL enhanced miR-451 loading by ∼12-fold relative to this pre-miR-451 scaffold baseline. Given that the pre-miR-451 backbone delivers 30-300 therapeutic siRNA copies per sEVs [18], our observed ∼12-fold enhancement *via* PMEVL suggests significant potential to increase RNA cargo delivery efficiency for artificial miRNA scaffold in sEVs. This potential is further supported by our demonstration that PMEVL enables efficient endogenous generation, packaging, and functional delivery of siRNAs using the distinct pri-miR-30a scaffold. The resulting improved payload capacity positions PMEVL-engineered sEVs as a promising alternative to clinically validated non-sEVs platforms like LNPs and GalNAc-siRNA conjugates. While LNPs (e.g., Patisiran) and GalNAc-siRNA systems (e.g., Inclisiran) achieve high hepatocyte delivery efficacy, they face limitations including hepatic confinement, immunogenicity risks, and dose-dependent toxicity [[45], [46], [47], [48]]. In contrast, PMEVL leverages the inherent biological advantages of sEVs, offering a potentially greater biocompatibility and targeting flexibility. Rigorous head-to-head comparisons with these established platforms remain essential to fully define PMEVL's therapeutic profile, including comparative efficacy, biodistribution, and safety.

PMEVL incorporates design and production features that may support its clinical translation. Its modular architecture allows flexible cargo customization: replacing the siRNA expression cassette within the miRNA backbone enables the generation of diverse siRNAs or the co-expression of multiple siRNAs, while replacing protein modules facilitates combined RNA/protein delivery. Key production-related characteristics include: (1) the miRNA-backbone-driven expression system permits the generation of stable producer cell lines, which helps ensure consistent sEVs cargo composition and yield across batches; (2) enhanced sEVs secretion and cargo-loading efficiency can reduce reliance on large-scale cell culture, thereby potentially lowering costs and improving scalability; and (3) no acute toxicity was observed in mice following systemic administration (though long-term safety profiles, such as immunogenicity upon repeated dosing, requires further evaluation). While fold enrichment effectively quantifies PMEVL's enhanced delivery capacity, future studies directly quantifying absolute siRNA copies per sEV would provide critical benchmarks for cross-platform efficiency comparisons. It also should be noted that, as with other sEVs-based platforms, optimizing large-scale isolation protocols to maintain purity and potency remains an important translational challenge.

In summary, we have developed the PMEVL platform—a robust and reproducible system for the simultaneous loading and delivery of small RNAs and proteins *via* sEVs. This work highlights its potential as a novel nanotherapeutic approach for diverse diseases.

## 4 Materials and methods

Extended methods details are provided in the Supplementary Materials.

### 4.1 Animal experiments

All animal procedures were approved by the Institutional Animal Care and Use Committee of Sun Yat-sen University (Protocol Nos. SYSU-IACUC-2024-000297 and SYSU-IACUC-2024-001483) and conducted in accordance with institutional guidelines.

For *in vivo* distribution studies, sEVs were labeled with DiR (AAT Bioquest, Catalog No. 22070) according to the manufacturer's instructions. Briefly, pelleted sEVs were incubated with 5 μM DiR at 37 °C for 30 min in the dark. Unbound dye was removed by ultracentrifugation at 120000×*g* for 70 min at 4 °C. Male BALB/c mice (6-week-old; Guangzhou GemPharmatech Co., Ltd.) received an intravenous injection of DiR-labeled sEVs (4 × 10^9^ particles in 100 μL PBS) or PBS vehicle control (100 μL). Whole-body fluorescence was monitored using an IVIS Spectrum system at 5, 24, 48, and 72 h post-injection. Mice were sacrificed at 72 h for ex vivo imaging of major organs (liver, lung, kidney, spleen, and heart).

For therapeutic evaluation, male C57BL/6 mice (6-week-old; Guangzhou GemPharmatech Co., Ltd.) received intravenous administrations of Myr-siPcsk9-sEVs or control Myr-siNC-sEVs (2.7 ✕ 10^10^ particles/mouse) every 3 days for 8 total doses. Mice were sacrificed 3 days after the final injection. Blood and tissues were collected for analysis.

### 4.2 Statistical analysis

The data were analyzed using GraphPad Prism 9.0 (GraphPad Software). All values are presented as mean ± standard error of the mean (SEM). Statistical significance was assessed using a two-sided unpaired *t-test* for comparisons between two groups or a *one-way analysis of variance (ANOVA)* test for comparisons among more than two groups. The following symbols indicate statistical significance: ns, not significant; ∗*p* < 0.05; ∗∗*p* < 0.01; ∗∗∗*p <* 0.001.

## CRediT authorship contribution statement

**Huijie Wang:** Formal analysis, Data curation, Conceptualization. **Xiaozhe Zhang:** Formal analysis, Data curation. **Yunjun Liang:** Formal analysis. **Zekai Zeng:** Methodology, Formal analysis. **Lianru Bi:** Software, Resources. **Yiying Yang:** Methodology, Formal analysis. **Jiajie Pan:** Software, Formal analysis. **Gang Dai:** Methodology. **Guifu Wu:** Writing – review & editing, Funding acquisition. **Wendong Fan:** Writing – review & editing, Writing – original draft, Conceptualization.

## Ethics approval and consent to participate

This study was approved by the Institutional Animal Care and Use Committee of Sun Yat-sen University (Protocol Nos. SYSU-IACUC-2024-000297 and SYSU-IACUC-2024-001483).

## Declaration of competing interests

The authors declare that they have no known competing financial interests or personal relationships that could have appeared to influence the work reported in this paper.
