# Supplementary material for: Robust loading and delivery of functional small RNAs via protein N-myristoylation-induced small extracellular vesicles
Source: Bioact Mater. 2026 Apr 13;63:422–38. doi: 10.1016/j.bioactmat.2026.04.012 (PMC13091954; doi:10.1016/j.bioactmat.2026.04.012)
Supplement: Multimedia component 1 [file mmc1.pdf]

Supplementary Materials

Robust Loading and Delivery of Functional Small RNAs *via* protein N-myristoylation-induced Small Extracellular Vesicles

This PDF file includes:

Materials and Methods

Supplemental Figures 1 to 16

Supplemental Tables 1 to 3

MATERIALS AND METHODS

Cell culture

Lenti-X 293T cells (Takara Bio, Catalog No. 632180) were cultured in Dulbecco's Modified Eagle's Medium (DMEM, high glucose; Gibco, Catalog No. C11995500BT) supplemented with 10% fetal bovine serum (FBS; Gibco, Catalog No. A31608-02), 1% penicillin/streptomycin (Gibco, Catalog No. 15140122), and 1% MEM non-essential amino acids (ThermoFisher Scientific, Catalog No. 11140050). Hepa1-6 cells (Procell, Catalog No. CL-0105) were maintained in high glucose DMEM (Gibco, Catalog No. C11995500BT) supplemented with 10% FBS and 1% penicillin/streptomycin. HUVECs were purchased from ScienCell (Catalog No. 8000) and grown in endothelial cell growth medium (ScienCell, Catalog No. 1001) containing 5% FBS, 1% endothelial cell growth supplement, and 1% penicillin/streptomycin. All cells were cultured at 37 °C in a humidified incubator with 5% CO_2.

Plasmid construction

To encapsulate exogenous, chemically synthesized siRNA and miRNA into sEVs, we generated the plasmids mCherry-Nluc-3 × FLAG, Myr(CHMP6)-mCherry-Nluc-3 × FLAG, and Myr(CHMP6)(G2A)-mCherry-Nluc-3 × FLAG as previously described[1], designated as CNF (control), Myr-CNF, and Myr(G2A)-CNF, respectively.

For endogenous miRNA loading, genes encoding pri-miR-200a-3p, pri-miR-125b-5p, and pri-miR-451 were PCR-amplified from lenti-X 293T cDNA and cloned into the 3′ UTR of either the CNF or Myr-CNF vector, generating miR-200a-3p, miR-125b-5p, miR-451, Myr-miR-200a-3p, Myr-miR-125b-5p, and Myr-miR-451 constructs. The pLV-GFP-HA and Myr-GFP-HA plasmids were constructed as previous described[1].

To evaluate pri-miRNA scaffolds for endogenous siRNA generation, sequences encoding siRNA targeting METTL3 within the backbone of pri-miR-30a, pri-let7b, pri-miR-92a, or pri-miR-23a were synthesized (Genewiz) and cloned into the 3′ UTR of the Myr-CNF vector, generating the corresponding siRNA-METTL3 constructs.

For endogenous siRNA loading, METTL3-, YAP-, EZH2-, or Pcsk9-targeting siRNA sequences within a pri-miR-30a backbone were PCR-amplified and assembled into the CNF or Myr-CNF vectors, producing siMETTL3, siYAP, siEZH2, siPcsk9, Myr-siMETTL3, Myr-siYAP, Myr-siEZH2, and Myr-siPcsk9 constructs. A negative control siRNA (siNC) was synthesized and cloned to generate Myr-siNC. For multiplexed gene targeting, three tandem pre-miR-30a backbones carrying YAP, EZH2, and METTL3 siRNA sequences were cloned into the CNF or Myr-CNF vectors to generate siYEM and Myr-siYEM constructs, respectively.

To assess the role of myristoylation in cargo loading, the myristoylation-deficient sequence from Myr(G2A)-CNF was inserted into the miR-200a-3p and siPcsk9 vectors, generating Myr(G2A)-miR-200a-3p and Myr(G2A)-siPcsk9 constructs. All siRNA sequences and primers are listed in Supplemental Tables 1 and 2.

To screen for optimal myristoylation peptides, cDNA sequences encoding the N-terminal 15-18 amino acids (representing the Myr-peptide) from 181 human myristoylated proteins were PCR-amplified and cloned in-frame to the N-terminus of the CNF reporter. Additionally, DNA sequences encoding peptides derived from FRS3, FYN, FGR, CABP1 (isoform L), and RNF11 were PCR-amplified and cloned into Myr-siPcsk9 plasmid to replace the Myr(CHMP6) sequence, generating the FRS3-siPcsk9, FYN-siPcsk9, FGR-siPcsk9, CABP1(isoform L)-siPcsk9, and RNF11-siPcsk9 constructs. Primers are listed in Supplemental Excel 2 and Supplemental Tables 2.

To identify proteins that interact with the Myr-peptide, we employed TurboID proximity labeling[2]. The TurboID-V5 sequences was synthesized (Genewiz) and cloned into the BamHI/SpeI-digested Myr-CNF vector, generating the Myr-TurboID construct. Primer sequences are provided in Supplemental Tables 2.

Lentivirus production and infection

Production: Recombinant lentiviruses were produced by co-transfecting lenti-X 293T cells with the target plasmid and helper plasmids psPAX2 (Addgene, Catalog No. 1226) and pMD2.G (Addgene, Catalog No. 12259) using Lipo293 transfection reagent (Beyotime, Catalog No. C0521). The culture medium was replaced 24 h post-transfection. Virus-containing supernatant was collected 48-72 h post-transfection, centrifuged at 3,200 ×g for 15 min, filtered through 0.45-μm polyethersulfone membrane (Merck Millipore), and concentrated using PEG8000 (Sigma, Catalog No. 81268) according to the manufacturer's protocol. Concentrated virus was pelleted (3,200 ×g, 15 min, 4 °C), aliquoted, and stored at −80 °C.

Infection: HUVECs at 60–70% confluent in 12-well plates were transduced with lentiviral particles in medium containing 5 μg/mL polybrene (Sigma-Aldrich, Catalog No. h9268). After 24 h, the medium was replaced with fresh complete growth medium. Cells were harvested for protein analysis 72 h post-transduction.

Transfection

Exogenous small RNAs: siRNA-METTL3 and a let-7i-5p mimic (Guangzhou Ruibo Biological) were used. To analyze small RNA delivery to cell culture supernatant, lenti-X 293T cells in 12-well plates (80%-90% confluence) were co-transfected with 2 μg plasmids DNA and 100 nM siRNA or miRNA mimic using 3.4 μL Lipo8000™ reagent (Beyotime, Catalog

No. C0533). Negative control siRNA (siNC) was purchased from Guangzhou RiboBio Co., Ltd. (Catalog No. siN0000001-1-5). For sEVs loading analysis, cells in 150-mm dishes were co-transfected with 24 μg plasmid DNA and 100 nM siRNA-METTL3 or let-7i-5p mimics using 29.4 μL Lipo8000™.

Endogenous small RNA: For supernatant analysis, lenti-X 293T cells in 12-well plates were transfected with 2 μg plasmids DNA using 3.4 μL Lipo8000™. For sEVs analysis, cells in 150-mm dishes were transfected with 24 μg plasmid DNA using 29.4 μL Lipo8000™.

Rescue experiment: In the in vitro rescue experiment, lenti-X 293T cells were transfected with the plasmid for 24 h, subsequently treated with DMSO (vehicle control), 0.5 mM AICAR (Beyotime, Catalog No. S1515), or 0.5 μM SCH772984 (Aladdin, Catalog No. S127539) for a total of 48 h.

Post-transfection processing: Following transfection, all cells were incubated in serum-free medium for 48 h. Supernatants were collected for extracellular RNA analysis or sEVs isolation, and cells were harvested for total intracellular RNA extraction or Western blot analysis.

sEVs isolation

sEVs were isolated by differential centrifugation[3]. Briefly, cell culture supernatant was sequentially centrifuged at 300 ×g for 10 min, 2000 ×g for 20 min, and 10,000 ×g for 30 min. The resulting supernatant was filtered (0.22-μm pore-size; Merck Millipore, Catalog No. SLGPR33RB) and subjected to ultracentrifugation at 120,000 ×g for 70 min at 4 °C using a Beckman Coulter Optima XE-100 Ultracentrifuge with a 70 Ti rotor. The sEVs pellet was resuspended in RIPA buffer (ThermoFisher Scientific, Catalog No. 89900) or phosphate-buffered saline (PBS; Servicebio, Catalog No. G4202).

sEVs characterization

Nanoparticle Tracking Analysis (NTA): Lenti-X 293T cells in 100-mm dishes were transfected with 12 μg plasmids DNA using 20.4 μL Lipo8000™. After 24 h of transfection, the culture medium was replaced with serum-free medium. Cells were then incubated for an additional 24 h before supernatant collection for sEVs isolation. At the time of supernatant harvest, the number of adherent cells was quantified using a ThermoFisher automated cell counter (Countess II). Purified sEVs were diluted in sterile, particle-free PBS to a concentration of 10^7–10^9 particles/mL. Size distribution and concentration were determined using a NanoSight NS300 instrument (Malvern Panalytical). For each sample, five independent 40-second videos were captured under continuous flow at 25 ± 1 °C. Results are reported as mean particle size (nm) ± standard deviation and particle concentration (particles/mL). sEVs yield

per cell was calculated by dividing the total number of isolated sEVs by the total number of lenti-X 293T cells at the time of harvest.

Transmission Electron Microscopy (TEM): A 5 μL aliquot of sEVs was applied to a copper grid for 2 min at 25 °C. Excess liquid was removed using Grade 1 qualitative filter paper. The grid was sequentially stained with 10 μL of 1% phosphotungstic acid solution for 1-2 min, followed by removal of excess stain. Morphological examination was performed using a JEM-1200EX transmission electron microscope (JEOL).

Western blot analysis

sEVs pellets, lenti-X 293T cells, and HUVECs were lysed on ice for 30 min in RIPA buffer (ThermoFisher Scientific, Catalog No. 89900) containing protease and phosphatase inhibitors (Beyotime, Catalog No. P1048). Protein concentrations were determined using the Pierce BCA Protein Assay Kit (ThermoFisher Scientific, Catalog No. 23227). Equal amounts of protein were denatured in 5× SDS loading buffer (Beyotime, Catalog No. P0015) at 98 °C for 10 min, separated by SDS-PAGE, and transferred on 0.45-μm polyvinylidene difluoride membranes (Merck Millipore, catalog no. IPVH00010). Membranes were blocked with 5% bovine serum albumin (Axygen, Catalog No. 43035) in 1× Tris-buffered saline containing 0.5% Tween-20 (TBST; Solarbio, Catalog No. T1082) for 1 h at 25 °C. Subsequently, the membranes were incubated with primary antibodies overnight at 4 °C, followed by horseradish peroxidase-conjugated secondary antibodies for 1 h at 25 °C. Protein bands were detected using a super-sensitive enhanced chemiluminescence detection reagent (Millipore, Catalog No. wbkls0100) and visualized using a chemiluminescence imaging system (GE Healthcare). Band intensities were quantified using ImageJ software and normalized to GAPDH. Antibody details are provided in Supplemental Table 3.

RNA quantification

Cell culture supernatants were collected 48 h post-transfection. After sequential centrifugation to remove debris and filtration (0.22-μm), extracellular RNA was isolated from 100 μL clarified supernatant or purified sEVs using TRIzol reagent. For intracellular RNA, cells were directly lysed in TRIzol. Isolated RNAs were reverse-transcribed using the RevertAid First Strand cDNA Synthesis Kit (ThermoFisher Scientific, Catalog No. K16225). RT-qPCR was performed with LightCycler 480 SYBR Green I Master Mix (Roche, Catalog, No. 4887352001) on a CFX96 Touch Real-Time PCR System (Bio-Rad). Relative intracellular miRNAs/siRNAs expression was calculated using the $2^{(-\Delta\Delta Cq)}$ method normalized to U6. Extracellular RNA levels in supernatants or sEVs were quantified and expressed relative to

intracellular levels for assessment of secretion or loading efficiency. Primer sequences are listed in Supplemental Table 2.

Cellular uptake assay

sEVs were fluorescently labeled with PKH67 (Sigma-Aldrich, Catalog No. MINI67) as described by the manufacturer. HUVECs or Hepa1-6 cells were seeded in 35-mm confocal dishes (JET BIOFIL, Catalog No. BDD012035). Labeled sEVs (1×10^9 particles per dish) were added to the cultures and incubated for 24 h at 37 °C. Cells were then washed thrice with PBS and fixed with 4% paraformaldehyde (Biosharp, Catalog No. BL539A) for 15 min. After washing the cells three times with PBS, the nuclei were counterstained with DAPI (Solarbio, Catalog No. C0065). Imaging was acquired using an LSM 780 laser scanning confocal microscope (ZEISS).

Vesicle RNA degradation assay

To confirm RNA encapsulation within sEVs, equal aliquots of engineered sEVs were treated with 1 μg/mL RNase A (TIANGEN, Catalog No. RT405) at 37 °C for 15 min, either in the presence or absence of 1% Triton-X-100 (Asegene, Catalog No. 43377) to permeabilize sEVs membranes. All reactions were terminated by adding 5 μL RNase Inhibitor (TIANGEN, Catalog No. DP418) for 15 min. Untreated sEVs served as a control. RNA was then extracted with TRIzol reagent and analyzed by RT-qPCR as described in Section 4.8.

Iodixanol density gradient fractionation

sEVs were further purified using an OptiPrep density gradient as described previously[4]. Briefly, a discontinuous gradient was prepared by diluting OptiPrep solution (Sigma-Aldrich, Catalog No. D1556) with sucrose working buffer (0.25 M, Tris HCl 10 mM, pH 7.4) to generate 5%, 10%, 20%, and 40% (w/v) iodixanol solutions. Gradients were assembled by sequential layering from bottom to top: 3 mL of 40% (w/v) iodixanol, 3 mL of 20% (w/v) iodixanol, 3 mL of 10% (w/v) iodixanol solution, 2.5 mL of 5% (w/v) iodixanol. The sEVs sample was loaded on top and centrifuged at 100,000 ×g for 18 h at 4 °C using an SW40Ti rotor. The top fraction (1 mL) was collected, diluted with 20 mL ice-cold PBS, and centrifuged at 100,000 ×g for 70 min at 4 °C. The resulting pellet was resuspended in PBS for downstream analyses.

Luciferase activity assay

Lenti-X 293T cells in 12-well plates (60%-70% confluence) were transfected with 2 μg of plasmid using Lipo8000™ Transfection Reagent. After 24 h, the medium was replaced with fresh complete medium. Supernatants were collected 48 h post-transfection and sequentially centrifuged at 300 ×g for 10 min, 2000 ×g for 10 min, and 10,000 ×g for 30 min at 4 °C. To degrade non-vesicle-associated NanoLuc luciferase, supernatants were treated with 100 μg/mL

proteinase K (Beyotime, Catalog No. ST533) at 37 °C for 2 h, followed by inactivation with 5 mM phenylmethylsulfonyl fluoride (PMSF; Beyotime, Catalog No. ST506). For measurement, 50 μL of treated supernatant was mixed with 50 μL of 1:50 diluted Nano-Glo Luciferase Assay substrate (Promega, Catalog No. N1120) in a white 96-well plate (Corning), and bioluminescence was immediately measured using a microplate reader (BioTek). For intracellular Nluc activity, cells were lysed in 1× Passive Lysis Buffer for 15 min at 25 °C, centrifuged at 12,000 ×g for 10 min, and the supernatant was assayed similarly. Cargo loading efficiency was expressed as the extracellular-to-intracellular (E/I) luminescence ratio (supernatant signal/lysate signal).

Immunofluorescence staining and histology

Paraffin-embedded liver sections were deparaffinized and rehydrated. After heat-mediated antigen retrieval, sections were blocked with 10% donkey serum at 37 °C for 1 h, followed by overnight incubation with a 1:400 dilution of an anti-LDLR primary antibody (Proteintech， Catalog No.10785). After washing with PBS, sections were incubated with Alexa Fluor™ 488 donkey anti-rabbit IgG (H+L) secondary antibody (ThermoFisher Scientific, Catalog No. A21206) for 1 h at 25 °C in the dark. Nuclei were counterstained with DAPI (Solarbio, Catalog No. C0060). Images were acquired using an automatic digital slide-scanning system (KF-FL-400, Ningbo). Relative mean fluorescence intensity (MFI) was analyzed using ImageJ software.

For histological analysis, tissues (heart, liver, spleen, lung, and kidney) were fixed in 4% paraformaldehyde, embedded in paraffin, sectioned, and stained with hematoxylin and eosin (H&E). Images were acquired using an automatic digital slide-scanning system (KF-PRO-120-H1, Ningbo).

Measurement of miRNA copy number per sEVs

Total RNA was isolated from a quantified number of sEVs (as determined by NTA). Synthetic miRNA standards (RiboBio) of known concentration were serially diluted. Both the diluted synthetic miRNA standards and the sEVs-derived RNA were reverse transcribed to cDNA. RT-qPCR was then performed on the resulting cDNA. A standard curve was generated by plotting the measured Cq values (Y-axis) against the log10 of the defined starting quantity (X-axis) of the synthetic miRNA standards. The copy number of target miRNAs in the sEVs-derived RNA samples was subsequently determined using this standard curve. The miRNA copy number per sEVs particle was determined by dividing the total miRNA copy number by the number of sEVs particles used for RNA isolation.

Cell Counting Kit-8 (CCK-8) assay

Cell viability was assessed using the Cell Counting Kit-8 (Solarbio, CA1210) according to the manufacturer's instructions. Lenti-X 293T cells were seeded in 24-well plates (40–50% confluence) and transfected with 1 μg plasmid using Lipo8000™. At 0, 24, and 48 h post-transfection, CCK-8 reagent was added to each well and incubated for 45 min at 37 °C in a humidified 5% CO_2 incubator. Absorbance at 450 nm was then measured.

Proteomic analysis

To investigate the molecular mechanism of PMEVL system, lenti-X 293T cells in 100-mm dishes were transfected with 10 μg of the Myr-TurboID plasmid using 20.4 μL of Lipo8000™. Forty-eight h post-transfection, cells were pulsed with 50 μM biotin (Sigma-Aldrich, Catalog No. B4501) in complete medium for 10 min at 37 °C. Cells were immediately placed on ice, washed three times with ice-cold DPBS (Gibco, Catalog No. C14190500BT) and harvested by scraping. Cell pellets were lysed in 300 μL of RIPA buffer supplemented with protease and phosphatase inhibitors. Cell lysates (300 μg protein) were incubated with 200 μL of streptavidin magnetic beads (Beyotime, Catalog No. P2151） overnight at 4 °C with rotation. Beads were washed stringently: twice with IP buffer (1 mL, 2 min; Catalog No. 87787, ThermoFisher Scientific), once with 1 M KCl (1 mL, 2 min), once with 0.1 M Na_2CO_3 (1 mL, 10 sec), and once with 2 M urea in 10 mM Tris–HCl buffer (pH 8.0) (1 mL, 10 sec), and twice again with IP buffer (1 mL, 2 min). Captured biotinylated proteins were eluted by boiling in 5× sample loading buffer. Eluted proteins were separated by SDS-PAGE and analyzed by Western blot to confirm successful enrichment. For mass spectrometry analysis, bead-bound proteins were digested with trypsin on-beads. The resulting peptides were desalted and analyzed using an Easy-nLC 1200 system coupled to a high-resolution mass spectrometer (ThermoFisher Scientific). Proteomic analysis was performed by OE Biotech Co., Ltd. (Shanghai, China).

Supplemental Figures with accompanying Figure legends

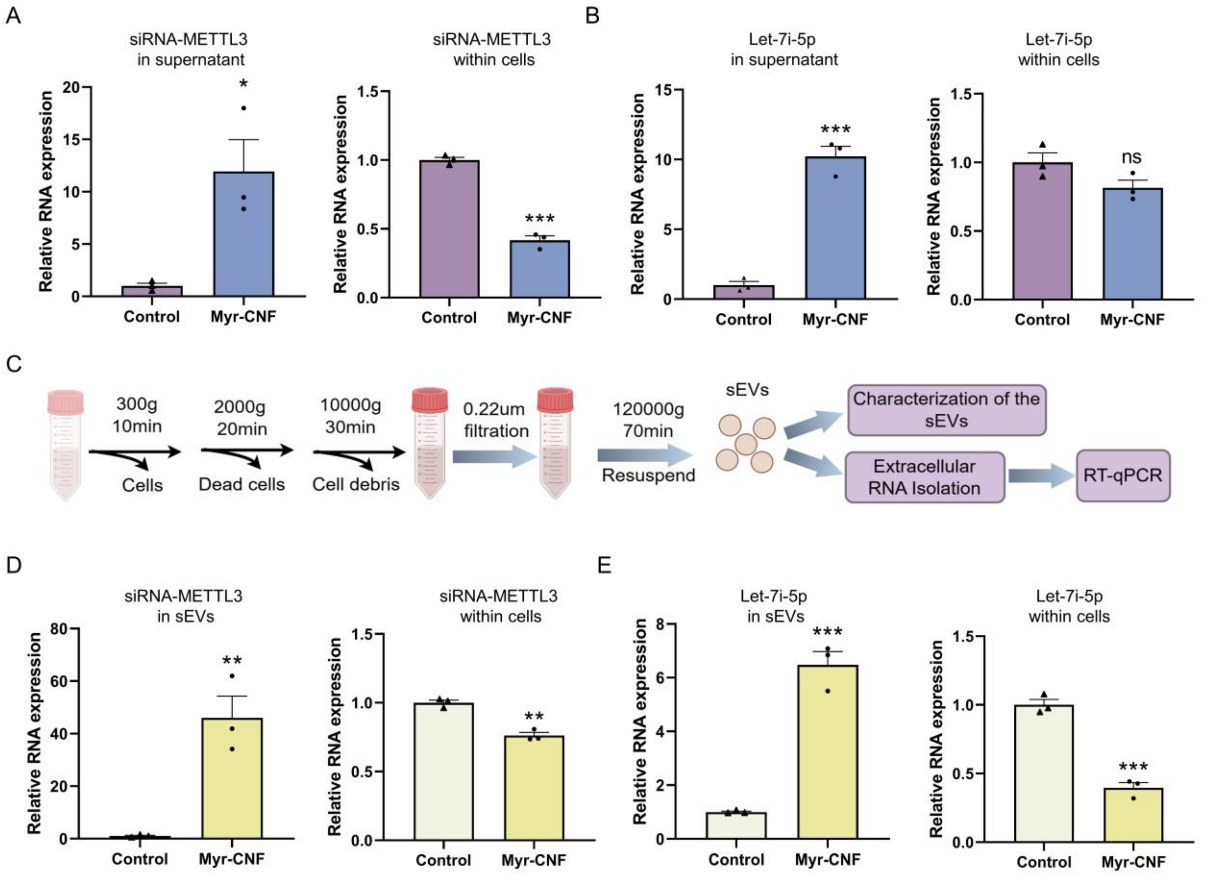

Figure S1. PMEVL platform enables active loading of exogenous siRNAs and miRNAs into small extracellular vesicles (sEVs). A and B. RT-qPCR analysis of exogenous chemically synthesized siRNA-METTL3(A) and let-7i-5p (B) in cell culture supernatants (left) and cells (right), corresponding to Figure 1B and 1C; n = 3. C. Schematic workflow for sEVs isolation and characterization. D and E. RT-qPCR analysis of exogenous siRNA-METTL3 (D) and let-7i-5p (E) in sEVs (left) and cells (right), corresponding to Figure 1I and 1J; n = 3. Data are presented as the means ± SEM. Statistical significance was determined by two-sided unpaired *t-test* (A-B and D-E). ns, not significant; * $P < 0.05$; ** $P < 0.01$; *** $P < 0.001$ versus control.

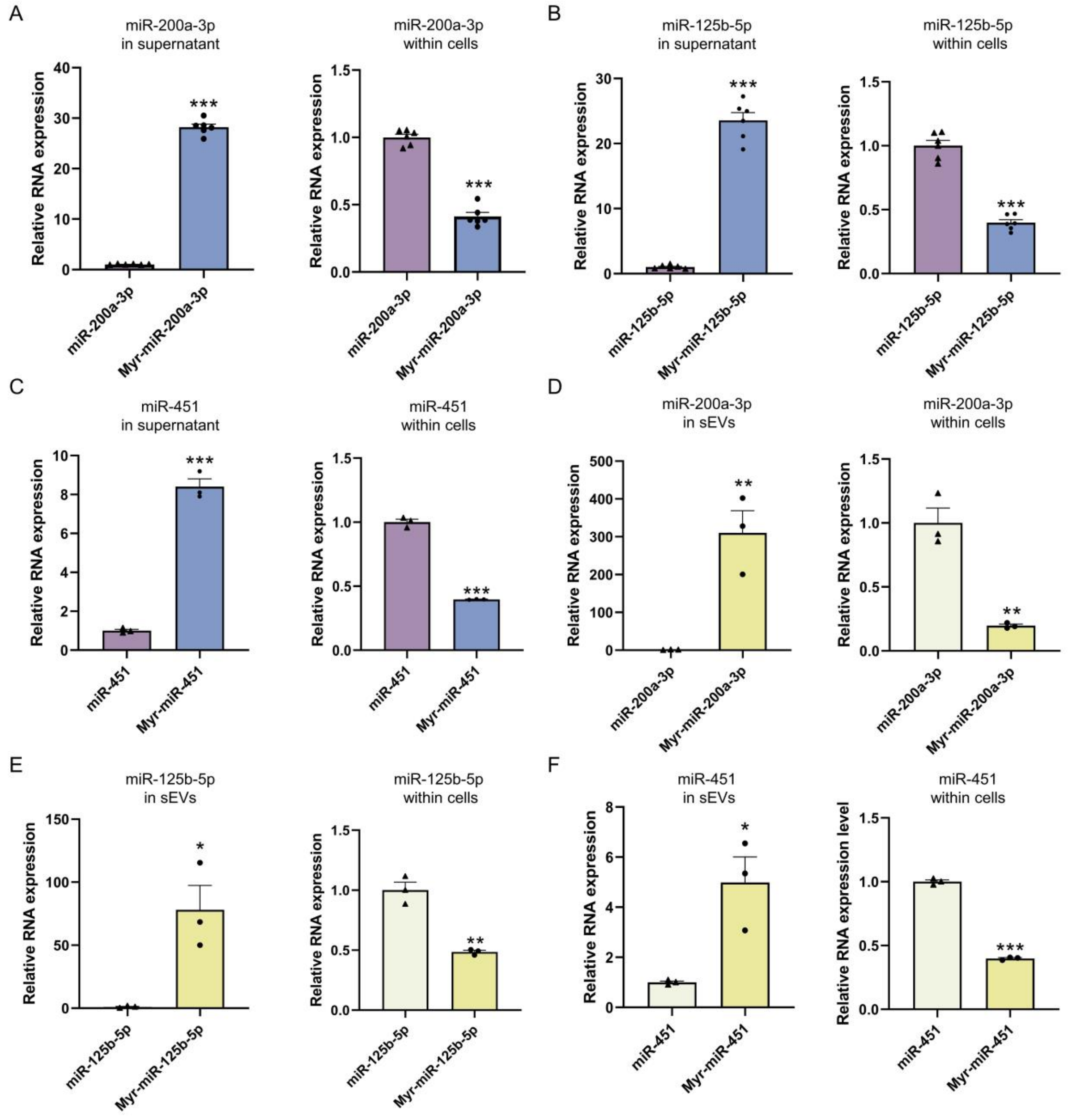

Figure S2. PMEVL significantly enriches endogenously expressed miRNAs in supernatants and sEVs. A-C. Endogenously synthesized miR-200a-3p (A), miR-125b-5p (B), miR-451 (C) levels quantified by RT-qPCR in culture supernatants (left) and cellular fractions (right), corresponding to Figure 2B-D; n = 3-6. D-F. Expression of endogenously synthesized miR-200a-3p (D), miR-125b-5p (E), miR-451(F) in sEVs (left) and cellular fractions (right), corresponding to Figure 2E-G; n = 3. Data are presented as the means ± SEM. Statistical significance was determined by two-sided unpaired *t-test.* * $P < 0.05$; ** $P < 0.01$; *** $P < 0.001$ versus control.

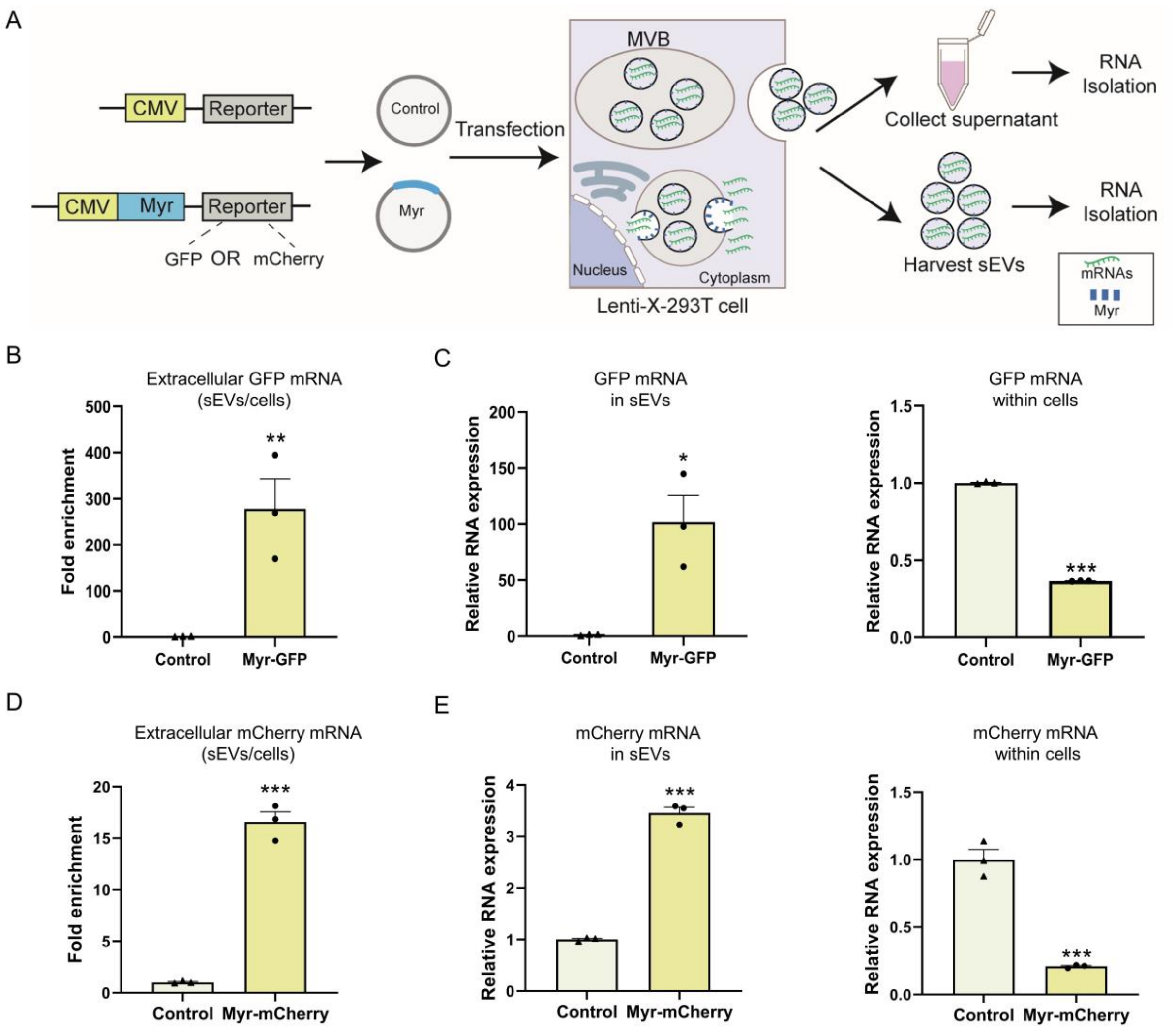

Figure S3. The PMEVL platform facilitates the loading of endogenously synthesized mRNAs into sEVs. A. Workflow for loading endogenously synthesized mRNAs into sEVs. B. Fold enrichment of GFP mRNA in sEVs. Enrichment was calculated as sEVs GFP mRNA level divided by GAPDH-normalized intracellular GFP mRNA level, relative to Control; n = 3. C. GFP mRNA expression levels in sEVs (left) and cells (right) measured by RT-qPCR; n = 3. D. Fold enrichment of mCherry mRNA in sEVs. Enrichment was calculated as sEVs mCherry mRNA level divided by GAPDH-normalized intracellular mCherry mRNA level, relative to Control; n = 3. E. mCherry mRNA expression levels in sEVs and cells measured by RT-qPCR; n = 3. Data are presented as the means ± SEM. Statistical significance was determined by two-sided unpaired *t-test* (B - E). * $P < 0.05$; ** $P < 0.01$; *** $P < 0.001$ versus control.

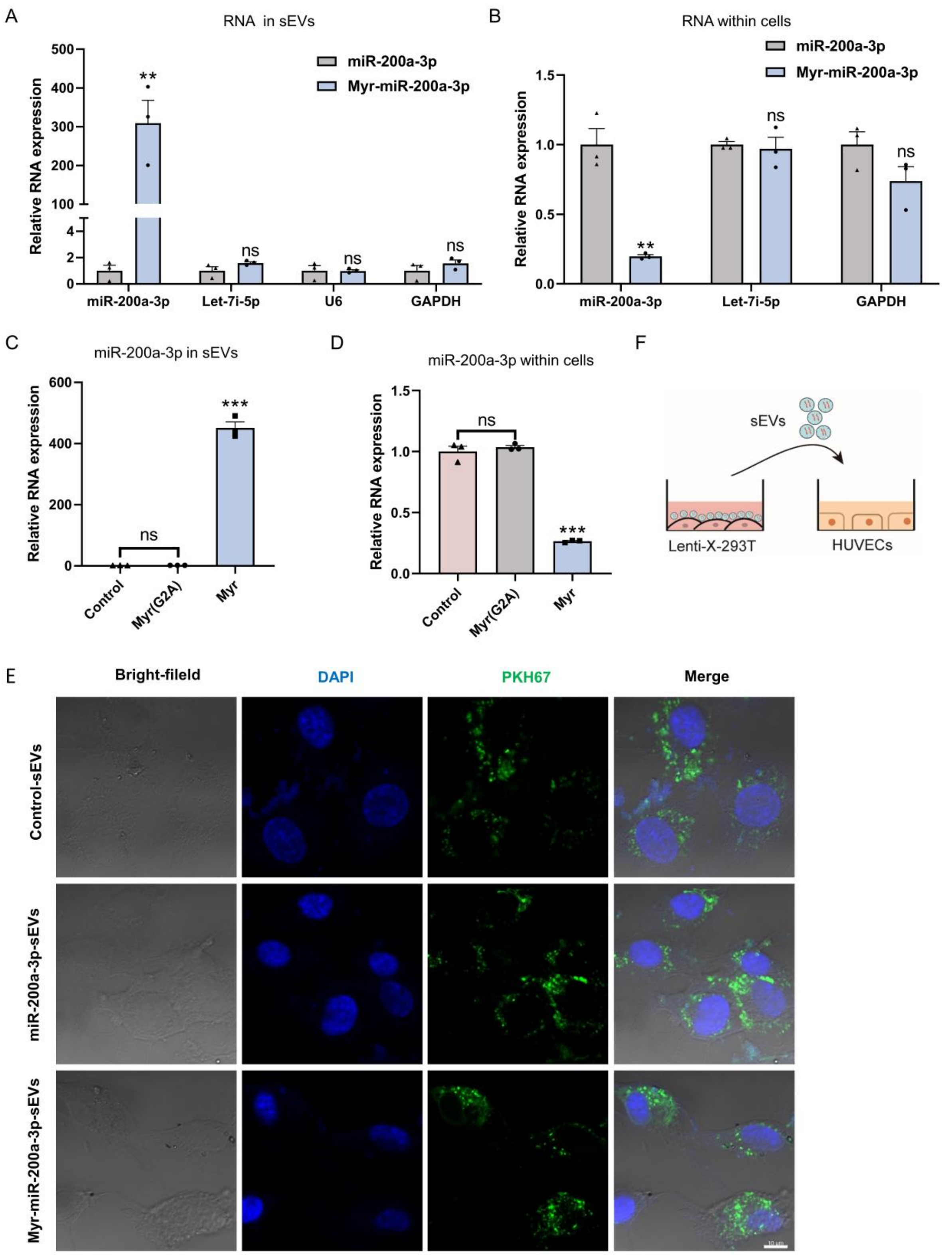

Figure S4. Protein N-myristoylation enables robust and specific miR-200a-3p loading. A and B. RT-qPCR analysis of miR-200a-3p, let-7i-5p, U6, and GAPDH mRNA expression in sEVs (A) and cells (B), corresponding to Figure 3A; n = 3. C and D. Expression levels of miR-200a-3p in sEVs (C) and cells (D) were determined by RT-qPCR, corresponding to Figure 3H;

n = 3. E. Confocal images of PKH67-labeled sEVs (green) internalized by HUVECs after 24 h. Nuclei: DAPI (blue). Scale bar: 10 μm. F. Functional delivery assay: sEVs-HUVEC co-culture schematic. Data are presented as the means ± SEM. Statistical significance was determined using two-sided unpaired *t-test* (A and B) or one-way *ANOVA* (C and D). ns, not significant; * $P < 0.05$; ** $P < 0.01$; *** $P < 0.001$ versus control.

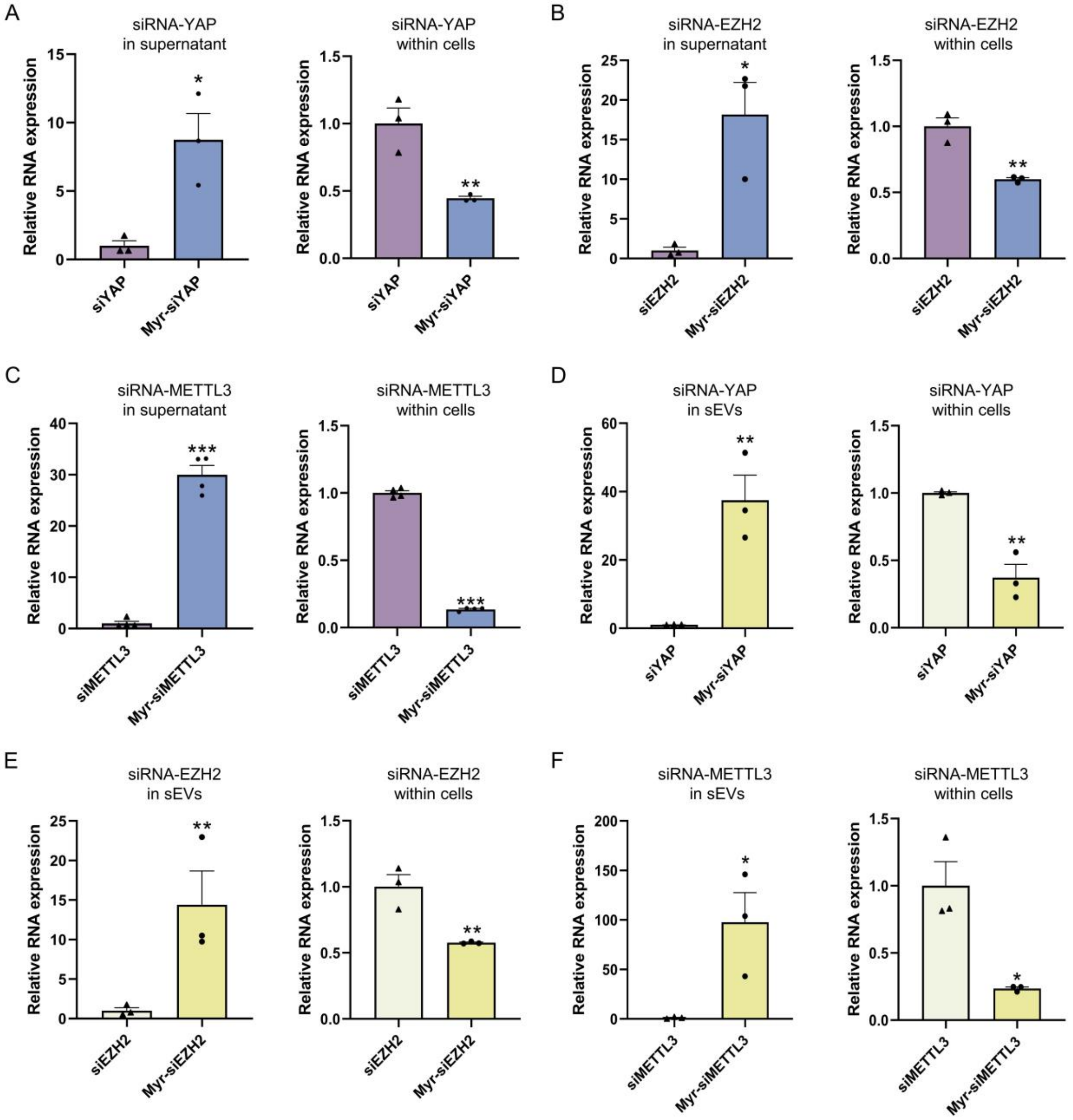

Figure S5. The PMEVL platform enables efficient loading of various endogenously synthesized siRNAs into sEVs. A-C. RT-qPCR analysis of endogenously produced siRNA-YAP (A), siRNA-EZH2 (B) and siRNA-METTL3 (C) in cell culture supernatants (left) and cells(right), corresponding to Figure 5B-D; n = 3. D-F. RT-qPCR analysis of siRNA-YAP (D), siRNA- EZH2 (E) and siRNA-METTL3 (F) in sEVs (left) and cells (right), corresponding to Figure 5E-G; n = 3. Data are presented as the means ± SEM. Significance was determined by two-sided unpaired *t-test*. * $P < 0.05$; ** $P < 0.01$; *** $P < 0.001$ versus control.

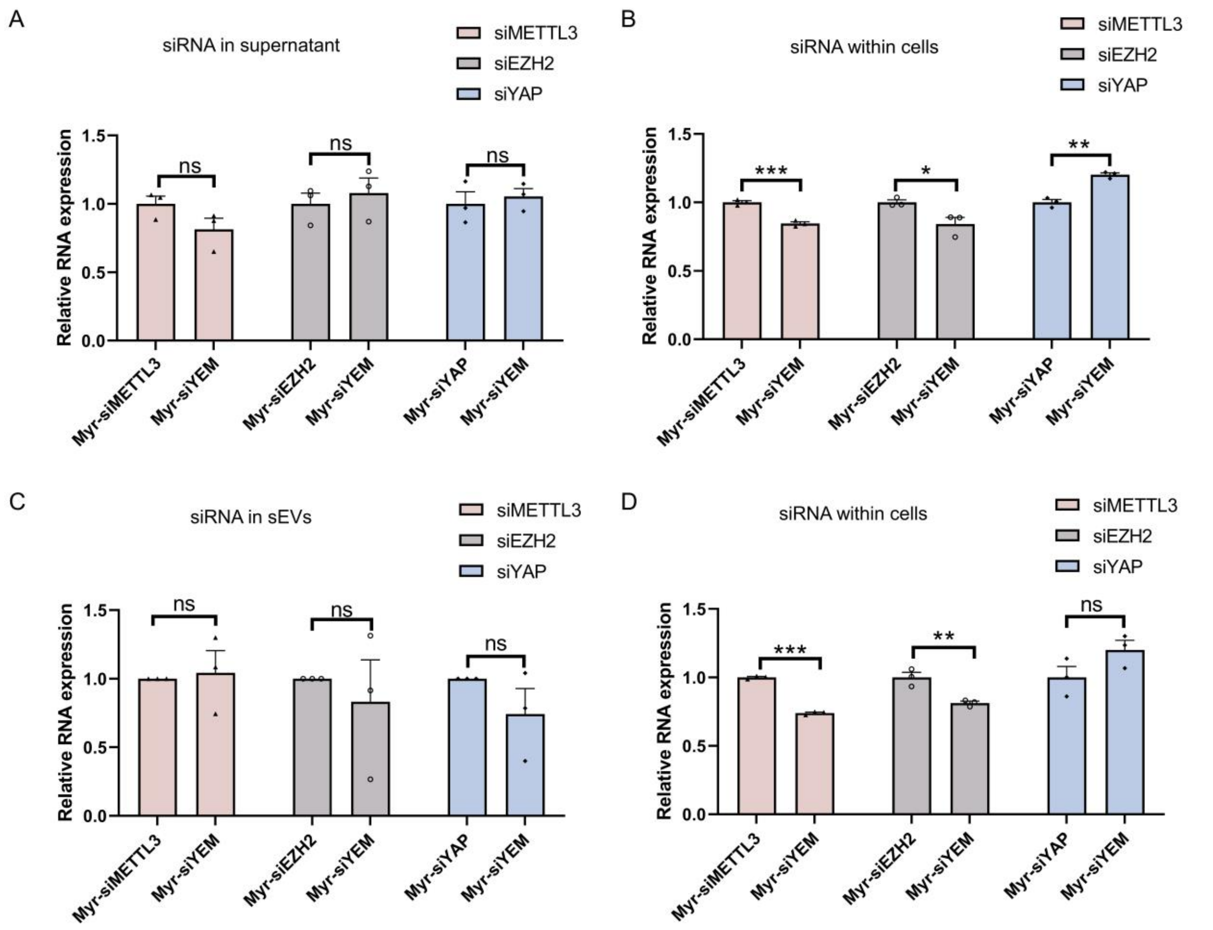

Figure S6. The PMEVL platform enables simultaneous loading of multiple siRNAs into sEVs. A and B. RT-qPCR analysis of siRNAs expression in cell culture supernatants (A) and cells (B), corresponding to Figure 5K; n = 3. C-D. RT-qPCR analysis of siRNAs expression in sEVs (left) and cells (right), corresponding to Figure 5L; n = 3. Data are presented as the means ± SEM. Statistical significance was determined by a two-sided unpaired t test. ns, not significant; * $P < 0.05$; ** $P < 0.01$; *** $P < 0.001$ versus control.

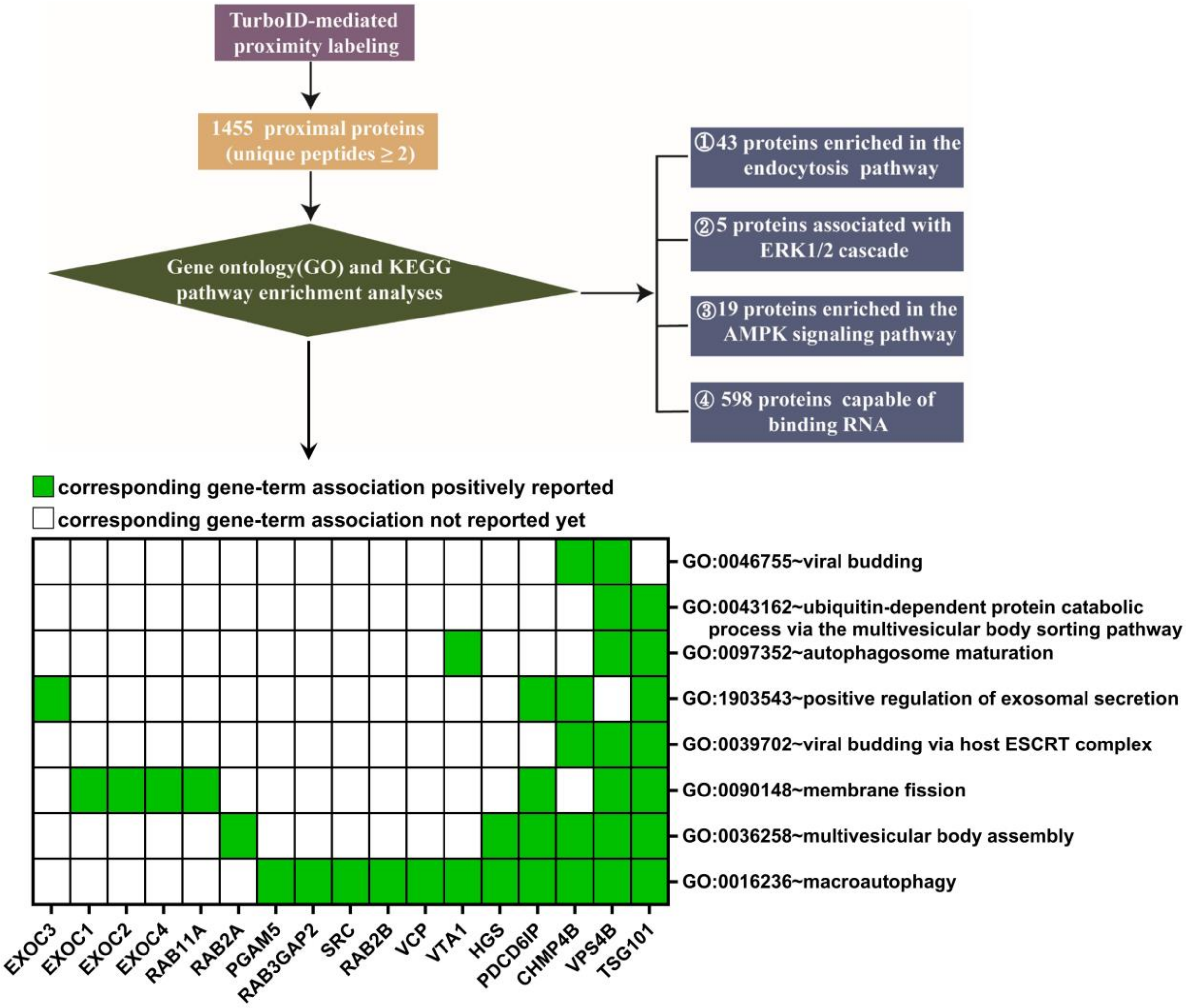

Figure S7. Gene Ontology (GO) and KEGG pathway enrichment analysis of the 1,455 high-confidence proximal proteins (≥2 unique peptides) identified by MS, performed using the DAVID Bioinformatics Resource.

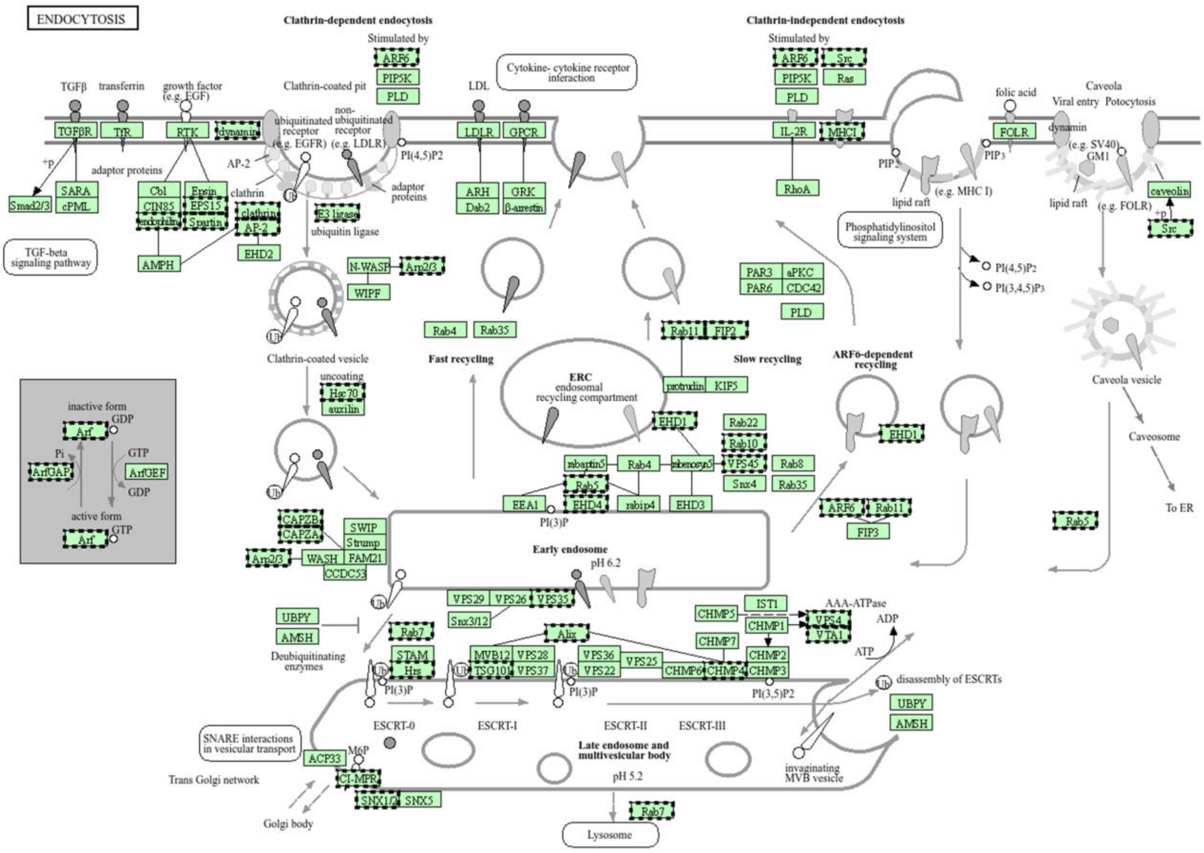

Figure S8. KEGG pathway enrichment analysis identifies endocytosis as a significantly enriched pathway among the proximal proteins of the Myr-peptide.

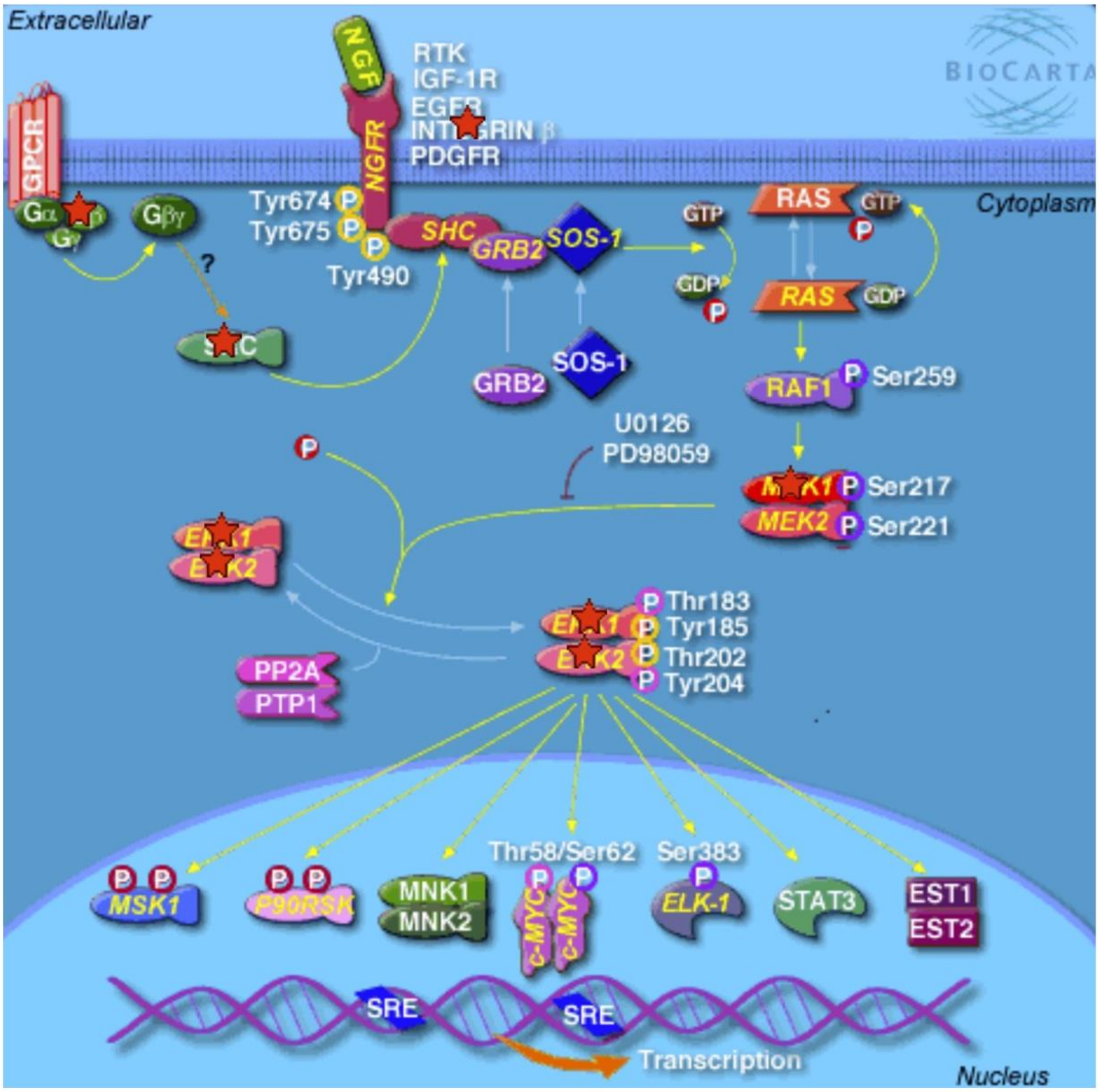

Figure S9. Enrichment analysis (BIOCARTA) reveals significant enrichment of the ERK1/ERK2 signaling pathway among proteins proximal to the Myr-peptide.

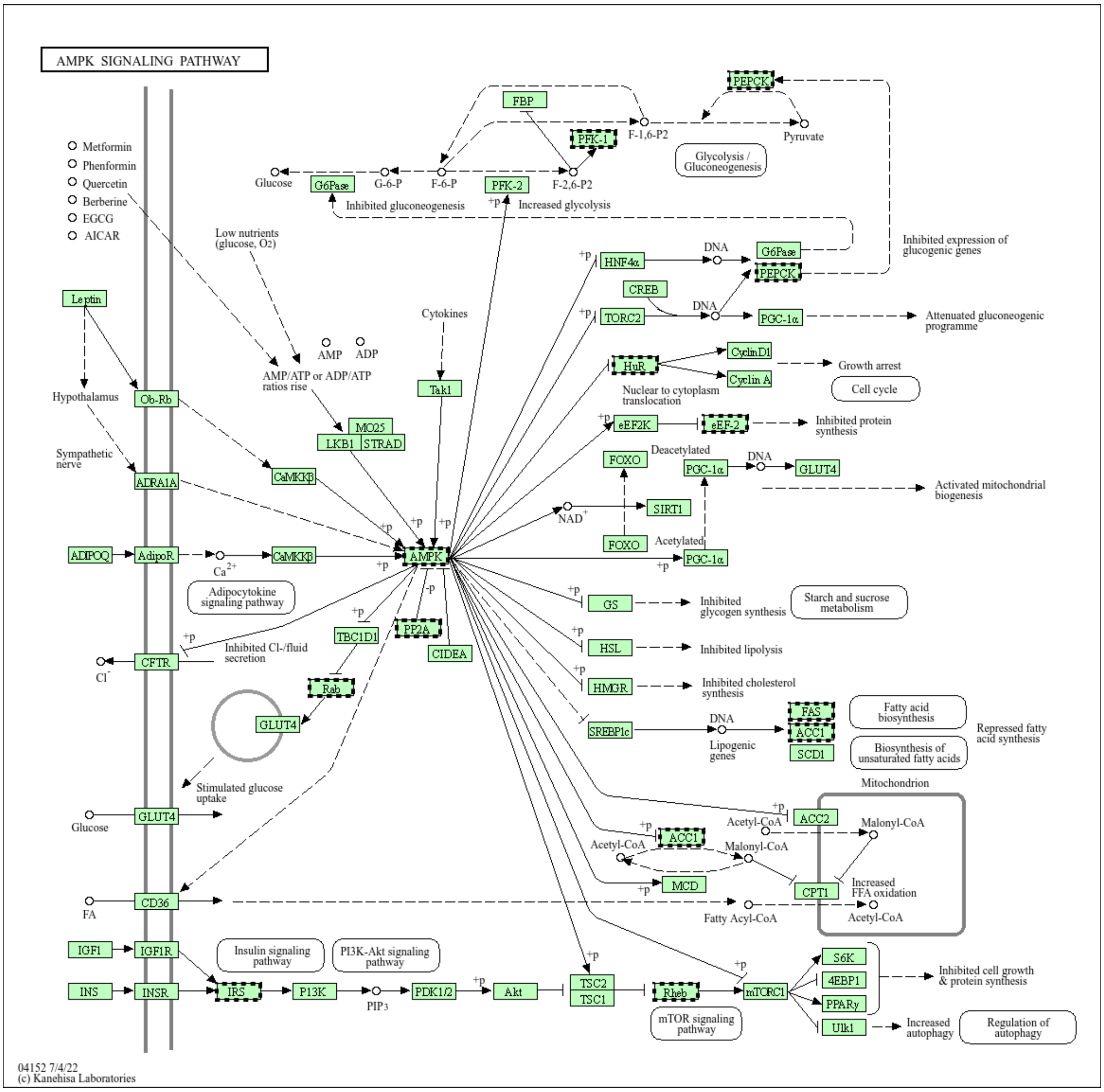

Figure S10. KEGG pathway enrichment analysis identifies AMPK as a significantly enriched pathway among the proximal proteins of the Myr-peptide.

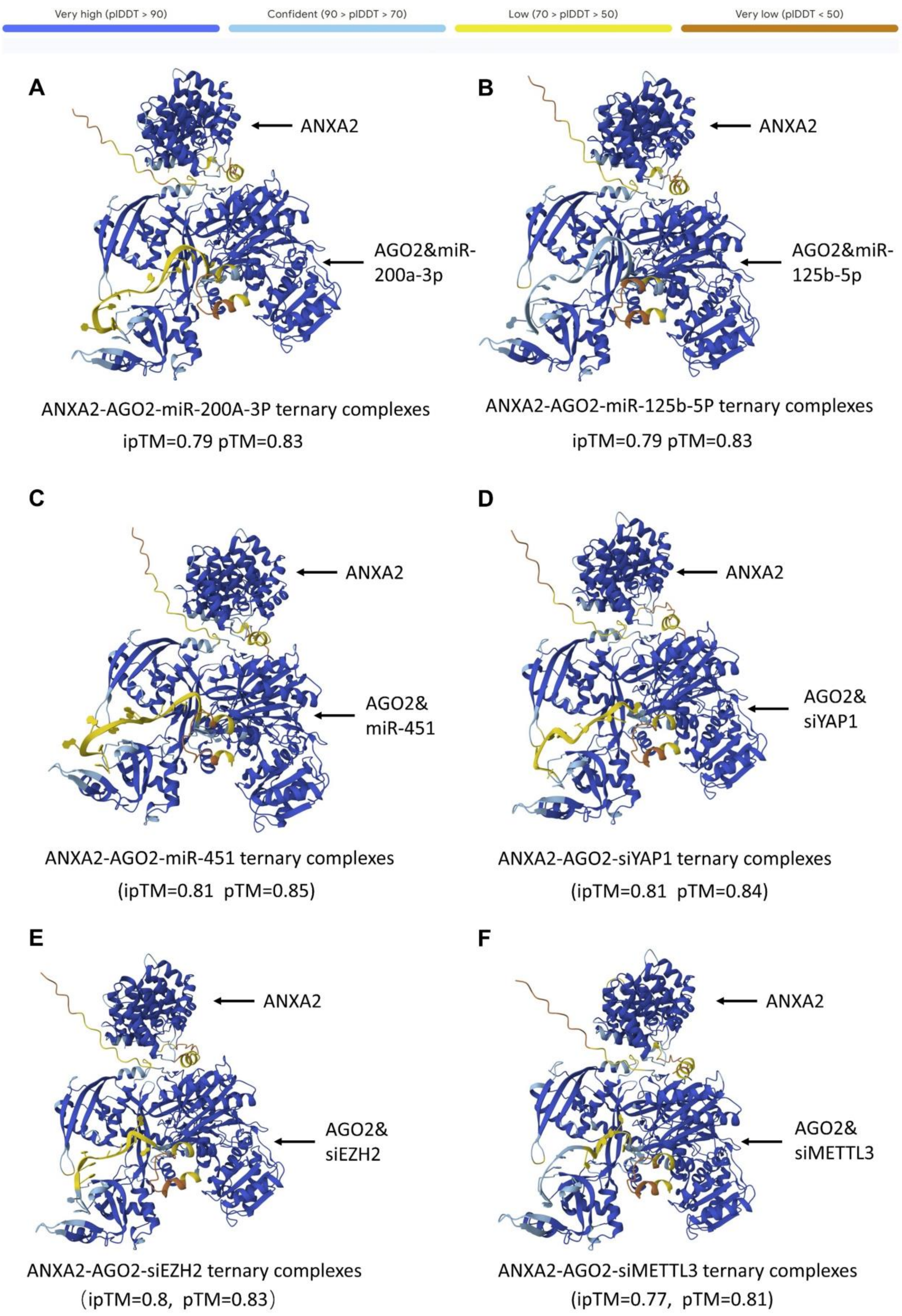

Figure S11. AlphaFold modeling predicts direct binding of ANXA2 to AGO2 and the

formation of ternary complexes with small RNAs. Predicted structural models of ANXA2-AGO2 complexes in association with (A) miR-200a-3p, (B) miR-125b-5p, (C) miR-451, (D) siRNA-YAP (siYAP), (E) siRNA-EZH2 (siEZH2), and (F) siRNA-METTL3 (siMETTL3) are shown, with the associated interface predicted TM-score (ipTM) and predicted TM-score (pTM) values indicated.

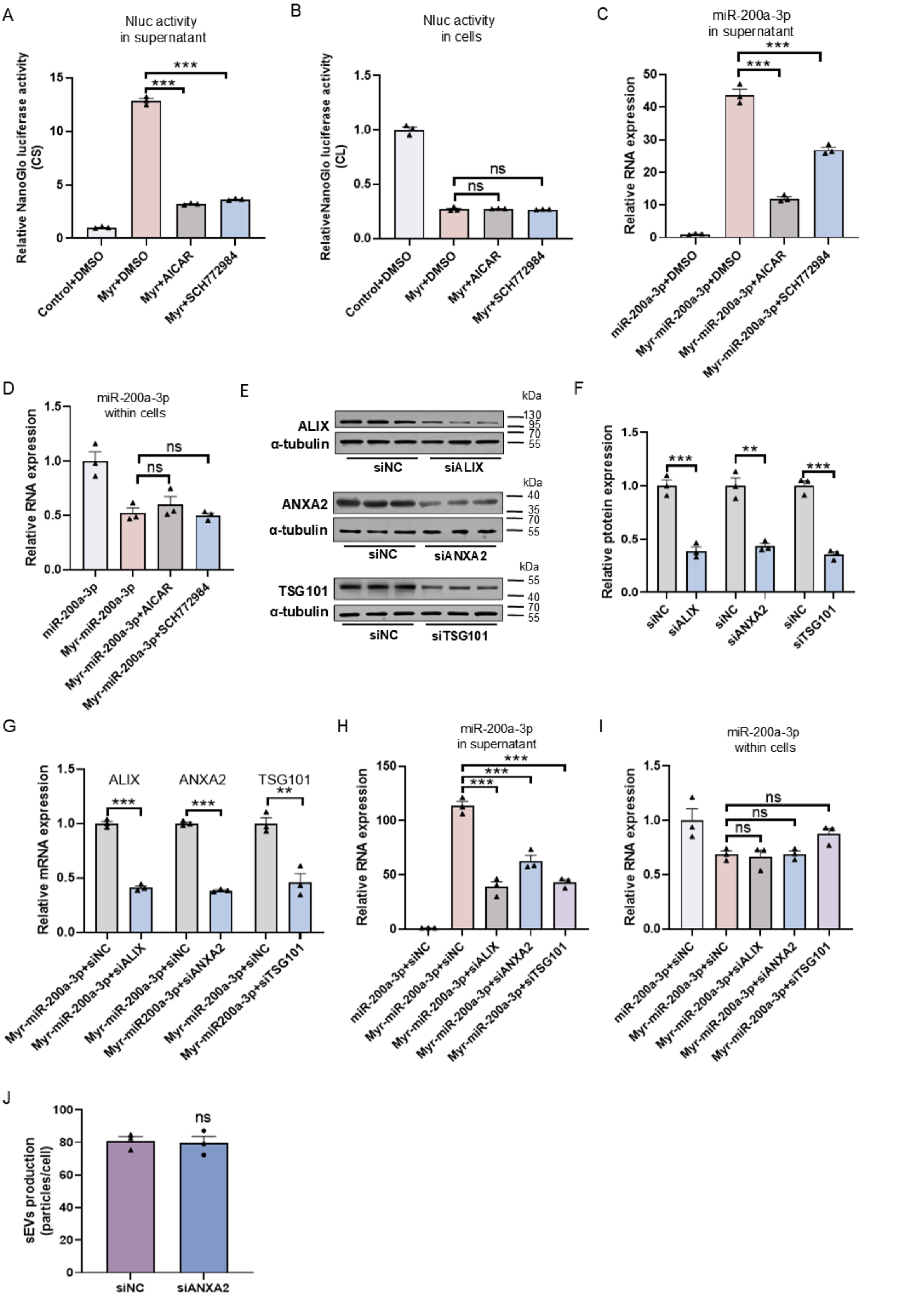

Figure S12. PMEVL promotes sEVs biogenesis and RNA loading by activating ERK1/2, inhibiting AMPK-autophagy, and recruiting ANXA2/ESCRT machinery. A-B. Nluc activity in the culture supernatants (CS) (A) and cell lysates (CL) (B) from lenti-X 293T cells

transfected with CNF (Control) or Myr-CNF (Myr) construct for 24 h, followed by 24 h treatment with: DMSO (vehicle control), AICAR (0.5 mM, AMPK activator), or SCH772984 (0.5 μM, ERK inhibitor); corresponding to Figure 6G; n = 3. C-D. RT-qPCR analysis of miR-200a-3p in cell culture supernatant (C) and cells (D), corresponding to Figure 6H; n = 3. E. Western blot analysis of TSG101, ALIX, and ANXA2 expression in lenti-X 293T cells transfected with control siRNA (siNC) or siRNA targeting ALIX (siALIX), ANXA2 (siANXA2) or TSG101 (siTSG101). F. Quantification of relative protein expression in E; n = 3. G. RT-qPCR analysis of ALIX, ANXA2 and TSG101; n = 3. H-I. RT-qPCR analysis of miR-200a-3p in cell culture supernatant (H) and cells (I), corresponding to Figure 6J; n = 3. J. sEVs production (particles/cell; NTA quantification) in lenti-X 293T cells transfected with siNC or siANXA2; n = 3. Data are presented as mean ± SEM. Statistical significance was determined by a two-sided unpaired *t-test* (F, G and J) or two-way *ANOVA* (A-D and H-I). * $P < 0.05$, ** $P < 0.01$, *** $P < 0.001$ versus control.

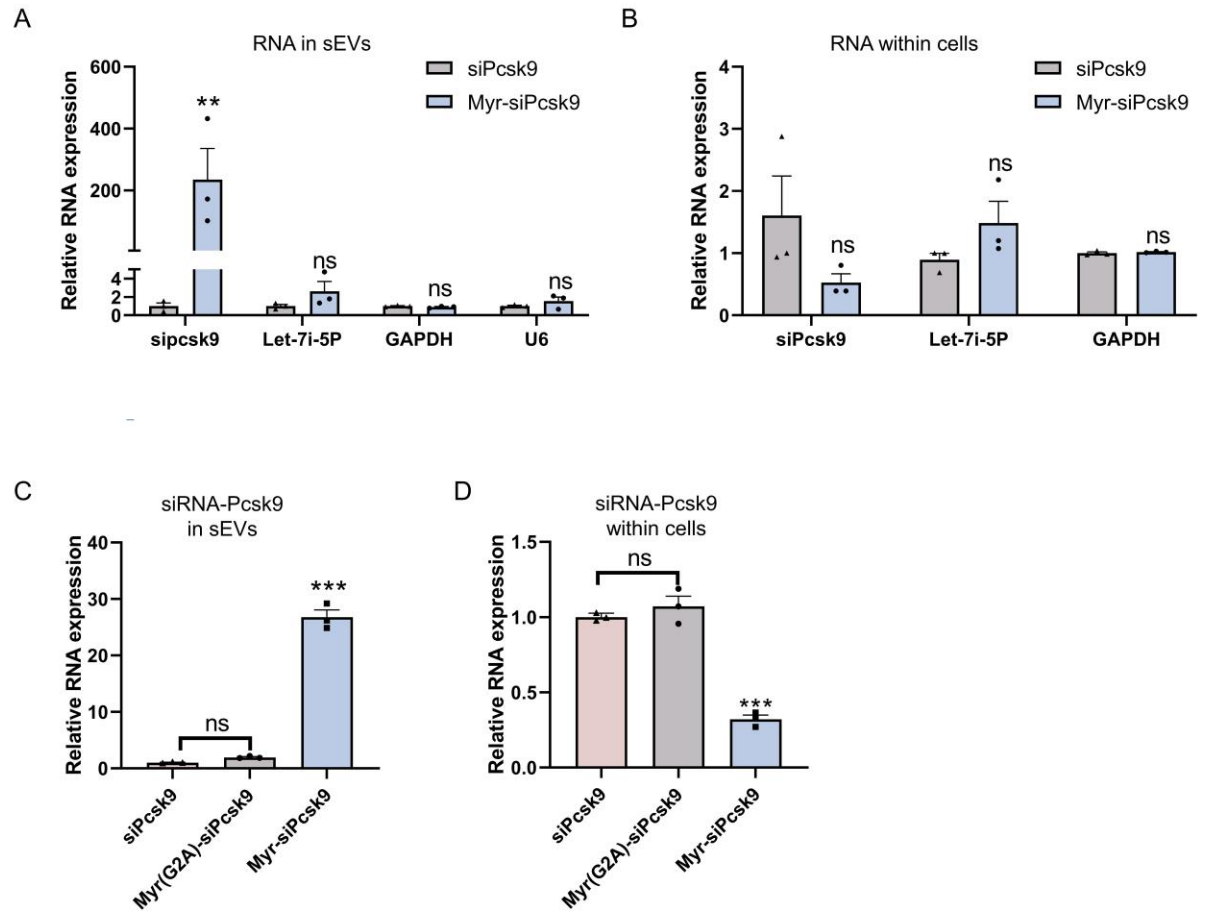

Figure S13. The PMEVL platform enables efficient, specific loading of endogenously synthesized siRNA-Pcsk9 into sEVs. A-B. Expression of siRNA-Pcsk9, let-7i-5p, U6, GAPDH mRNA in sEVs (A) and cells (B) by RT-qPCR, corresponding to Figure 7A; n = 3. C-D. Expression of si-Pcsk9 in sEVs (C) and cells (D) by RT-qPCR, corresponding to Figure 7E; n = 3. Data are presented as the means ± SEM. Statistical significance was determined by a two-sided unpaired *t-test* (A-B), or one-way *ANOVA* (C-D). ns, not significant; * $P < 0.05$; ** $P < 0.01$; *** $P < 0.001$ versus control.

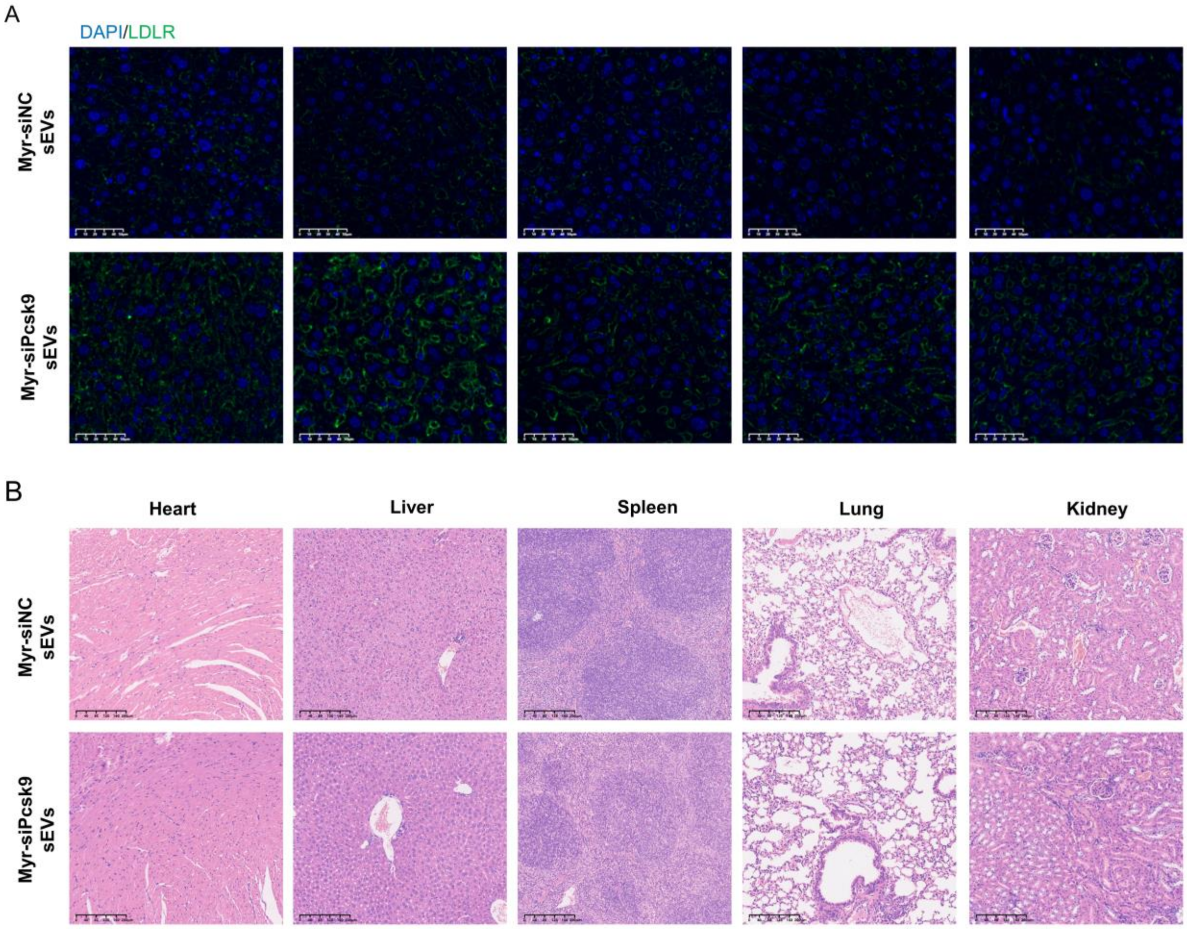

Figure S14. Immunofluorescence and hematoxylin and eosin (H&E) staining of major organs in C57BL/6 mice. A. The immunofluorescence analysis of LDLR expression in liver tissue (corresponding to Figure 8H). LDLR (green), DAPI (blue). Scale bar: 50 μm. B. Representative H&E-stained sections of heart, liver, spleen, lung and kidney. Scale bar: 200 μm.

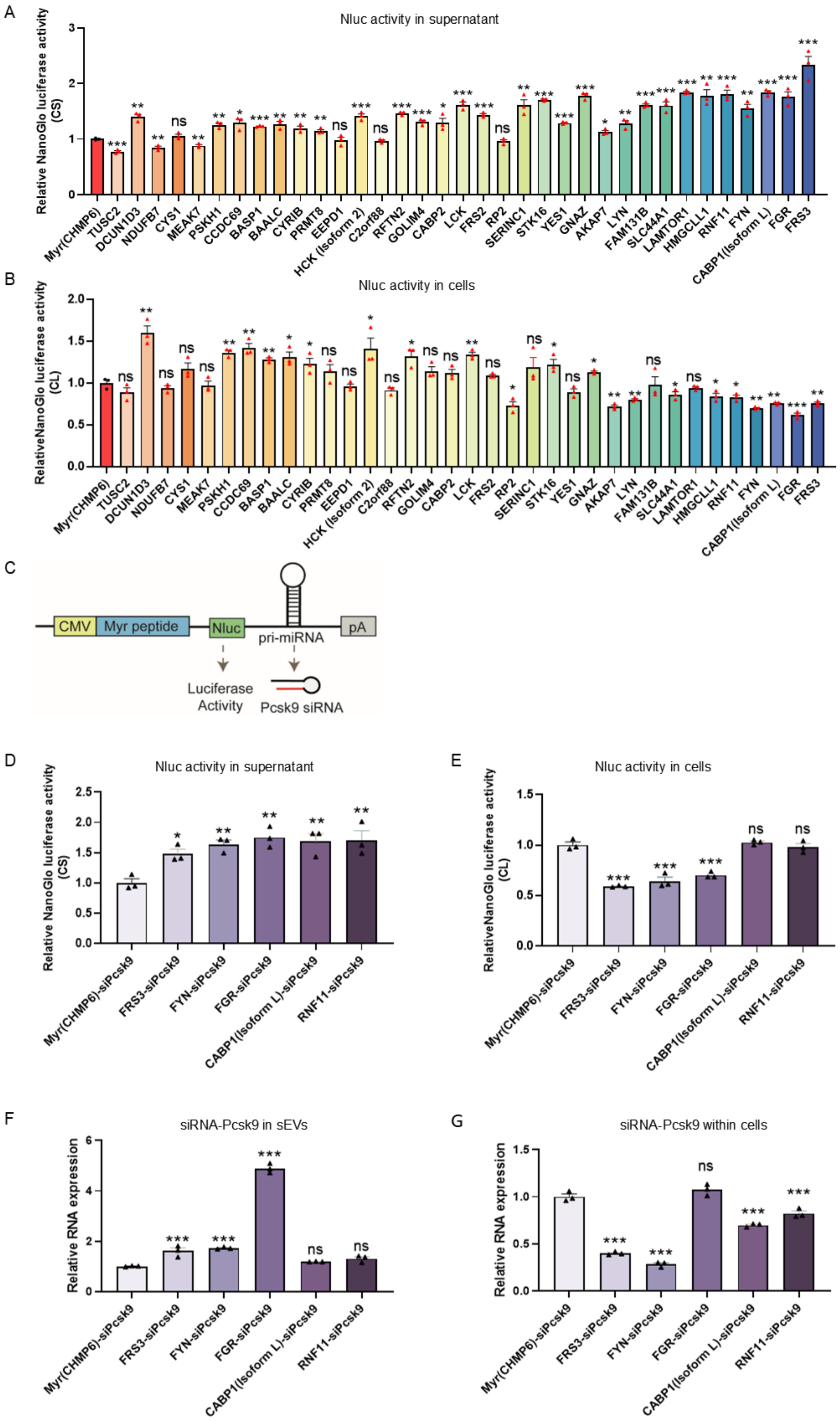
A
Nluc activity in supernatant
Relative NanoGlo luciferase activity (CS)
Myr(CHMP6) TUSC2 DCUN1D3 NDUFB7 CYS1 MEAK7 PSKH1 CCDC69 BASP1 BAALC CYRIB PRMT8 EEPD1 HCK (Isoform 2) C2orf88 RFTN2 GOLIM4 CABP2 LCK FRS2 RP2 SERINC1 STK16 YES1 GNAZ AKAP7 LYN FAM131B SLC44A1 LAMTOR1 HMGCLL1 RNF11 FYN CABP1(Isoform L) FGR FRS3
B
Nluc activity in cells
RelativeNanoGlo luciferase activity (CL)
C
CMV
Myr peptide
Nluc
pri-miRNA
pA
Luciferase Activity
Pcsk9 siRNA
D
Nluc activity in supernatant
Relative NanoGlo luciferase activity (CS)
Myr(CHMP6)-siPcsk9 FRS3-siPcsk9 FYN-siPcsk9 FGR-siPcsk9 CABP1(Isoform L)-siPcsk9 RNF11-siPcsk9
E
Nluc activity in cells
RelativeNanoGlo luciferase activity (CL)
F
siRNA-Pcsk9 in sEVs
Relative RNA expression
G
siRNA-Pcsk9 within cells
Relative RNA expression

Figure S15. Screening of peptides derived from putative human N-myristoylated proteins to optimize cargo loading capacity of PMEVL. A-B. Nluc activity in the culture supernatants (CS) (A) and cell lysates (CL) (B) form sEVs-producing cells, corresponding to Figure 9B; n = 3. C. Engineering strategy for co-loading siRNAs and proteins *via* Myr-peptides. D-E: Nluc activity in the culture supernatants (CS) (D) and cell lysates (CL) (E) from sEVs-producing cells, corresponding to Figure 9D; n = 3. F-G. Expression levels of siRNA-Pcsk9 in sEVs (F) and cells (G) determined by RT-qPCR, corresponding to Figure 9E; n = 3. Data are presented as the means ± SEM. Statistical significance was determined by two-sided unpaired *t-test* (A-B and D-G). ns, not significant; * $P < 0.05$; ** $P < 0.01$; *** $P < 0.001$ versus control.

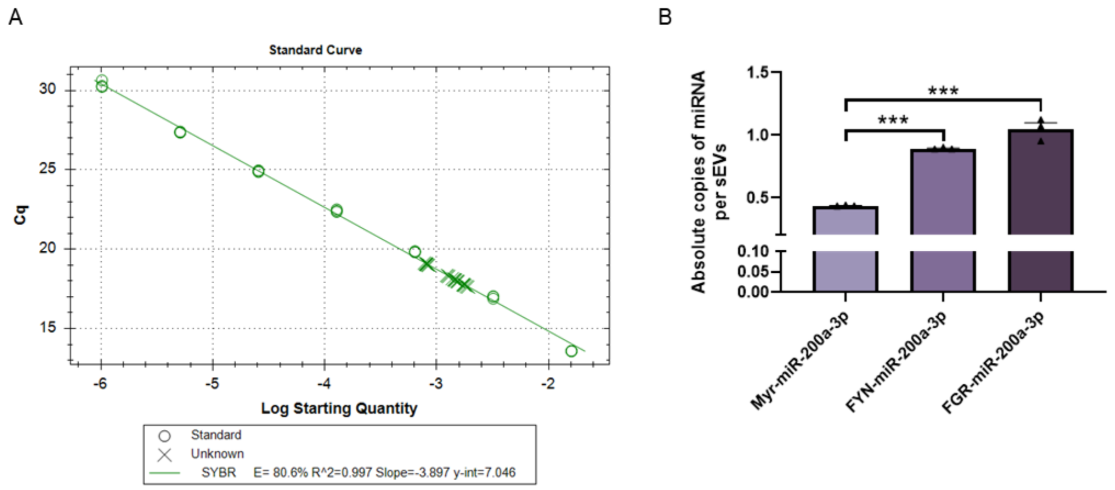

Figure S16. Absolute quantification of miR-200a-3p copies per sEVs particle using optimized PMEVL. A. Standard curve for absolute quantification of miR-200a-3p. B. miR-200a-3p loading efficiency per sEVs particle mediated by N-myristoylated peptides Myr(CHMP6), FYN, and FGR. Values represent absolute miRNA copies per sEVs particle normalized to sEVs counts determined by Nanoparticle Tracking Analysis, n = 3. Data are presented as the means ± SEM. Statistical significance was determined by one-way *ANOVA* (B). ns, not significant; * $P < 0.05$; ** $P < 0.01$; *** $P < 0.001$.

Supplemental tables

Supplemental Table 1. siRNAs sequences

Name	Sense Strand (5’ -3’)	Antisense Strand (5’ -3’)
METTL3 siRNA	C.A.G.U.G.G.A.U.C.U.G.U.U.G.U.G.A.U.A.dTdT	U.A.U.C.A.C.A.A.C.A.G.A.U.C.C.A.C.U.G.dTdT
YAP1 siRNA	CCACCAAGCUAGAUAAAGA dTdT	UCUUUAUCUAGCUUGGUGG dTdT
EZH2 siRNA	CAGAAGAACUAAAGGAAAA dTdT	UUUUCCUUUAGUUCUUCUG dTdT
Pcsk9 siRNA#3	C.C.A.A.A.G.A.C.G.U.C.A.U.C.A.A.C.A.U. dTdT	A.U.G.U.U.G.A.U.G.A.C.G.U.C.U.U.U.G.G. dTdT
ANXA2 siRNA	GGU CUG AAU UCA AGA GAA A dTdT	UUU CUC UUG AAU UCA GAC C dTdT
TSG101 siRNA	C.C.G.U.U.U.A.G.A.U.C.A.A.G.A.A.G.U.A.dTdT	U.A.C.U.U.C.U.U.G.A.U.C.U.A.A.A.C.G.G.dTdT
ALIX siRNA	G.G.C.A.C.A.G.G.C.U.C.A.A.G.A.A.G.U.A. dTdT	U.A.C.U.U.C.U.U.G.A.G.C.C.U.G.U.G.C.C. dTdT

Supplemental Table 2. Primers

Name	Sense Primer (5’ -3’)	Antisense Primer (5’ -3’)
Primers used to construct plasmids		
miR200a-3p	ATCCAGAGGTTGATTGTCGACgccctcacccgtctgctggc	gagcggATGAGTCGACcctgtccggtcccggcacca
Myr-miR200a-3p	ATCCAGAGGTTGATTGTCGACgccctcacccgtctgctggc	cGGATCCTGAGTCGACcctgtccggtcccggcacca

miR125b-5p	ATCCAGAGGTTGATTGTCGACcctcagaggtatactcaatcacctcagac	gagcggATGAGTCGACccataccacctgtttgttgcatct
Myr-miR125b-5p	ATCCAGAGGTTGATTGTCGACcctcagaggtatactcaatcacctcagac	cGGATCCTGAGTCGACccataccacctgtttgttgcatct
miR-451	TAATCCAGAGGTTGATTGTCGACcctacccccaatcccacgc	gagcggATGAGTCGACgcacccccagctctggag
Myr-miR-451	TAATCCAGAGGTTGATTGTCGACcctacccccaatcccacgc	cGGATCCTGAGTCGACgcacccccagctctggag
si-METTL3	AATCCAGAGGTTGATTGTCGACCCCTTGAAGTCCGAGGCAGCA	gagcggATGAGTCGACTGCTGTTGACAGTGAGCGAAcagt
Myr-siMETTL3	AATCCAGAGGTTGATTGTCGACCCCTTGAAGTCCGAGGCAGCA	cGGATCCTGAGTCGACTGCTGTTGACAGTGAGCGAAcagt
siYAP Primer pairs 1	AATCCAGAGGTTGATTGTCGACCCCTTGAAGTCCGAGGCAGCA	CAGTGAAGCCACAGATGTGtctttatctagcttggtggTCTGCCTGCTGCCTCGGACTT
siYAP Primer pairs 2	ataaagaCACATCTGTGGCTTCACTGtctttatctGAagcttggtggTTCGCTCACTGT	gagcggATGAGTCGACTGCTGTTGACAGTGAGCGAAccaccaagc
Myr-siYAP Primer pairs 1	AATCCAGAGGTTGATTGTCGACCCCTTGAAGTCCGAGGCAGCA	CAGTGAAGCCACAGATGTGtctttatctagcttggtggTCTGCCTGCTGCCTCGGACTT
Myr-siYAP Primer pairs 2	ataaagaCACATCTGTGGCTTCACTGtctttatctGAagcttggtggTTCGCTCACTGT	cGGATCCTGAGTCGACTGCTGTTGACAGTGAGCGAAccaccaagc
siEZH2 Primer pairs 1	AATCCAGAGGTTGATTGTCGACCCCTTGAAGTCCGAGGCAGCA	CAGTGAAGCCACAGATGTGttttccttagttcttctgTCTGCCTGCTGCCTCGGACTT

siEZH2 Primer pairs 2	aggaaaaCACATCTGTGGCTTCACTGttttcctttGAagttcttctgTTCGCTCACTGT	gagcggATGAGTCGACTGCTGTTGACAGTGAGCGAAcagaagaactTC
Myr-siEZH2 Primer pairs 1	AATCCAGAGGTTGATTGTCGACCCCTTGAAGTCCGAGGCAGCA	CAGTGAAGCCACAGATGTGttttcctttagttcttctgTCTGCCTGCTGCCTCGGACTT
Myr-siEZH2 Primer pairs 2	aggaaaaCACATCTGTGGCTTCACTGttttcctttGAagttcttctgTTCGCTCACTGT	cGGATCCTGAGTCGACTGCTGTTGACAGTGAGCGAAcagaagaactTC
si-YEM Primer pairs 1	AGCGCTGTCGACCCCTTGAAGTCCGAGG	gTggAggCggctcgagcggATAAGTCGACTGCTGTTGACAGTGAGC
si-YEM Primer pairs 2	AGTCGACTCATccgctcgagACCGGTCCCTTGAAGTCCGAGGCAG	AGGGGTCGACAGCGCTTGCTGTTGACAGTGAGCGAAcaga
Myr-siYEM Primer pairs 1	AGCGCTGTCGACCCCTTGAAGTCCGAGG	gTggAggCggctcgagcggATAAGTCGACTGCTGTTGACAGTGAGC
Myr-siYEM Primer pairs 2	AGTCGACTCATccgctcgagACCGGTCCCTTGAAGTCCGAGGCAG	AGGGGTCGACAGCGCTTGCTGTTGACAGTGAGCGAAcaga
Myr-siPsk9 Primer pairs 1	AATCCAGAGGTTGATTGTCGACCCCTTGAAGTCCGAGGCAGCAGGCA	CAGTGAAGCCACAGATGTGATGTTGATGACGTCTTTGGTCTGCCTGCTGCCTCGGACTT
Myr-siPsk9 Primer pairs 2	CAACATCACATCTGTGGCTTCACTGATGTTGATGGAACGTCTTTGGTTCGCTCACTGT	cGGATCCTGAGTCGACTGCTGTTGACAGTGAGCGAACCAAAGACG
siPcsk9	AGACACCGACTCTAGAggccgccaccATGGTGAGCAAGGGCGAGGAGGATAA	ActAccTccTccgccACTAGACTTGTACAGCTCGTCCATGCCG
CABP1(Isoform L)-siPcsk9	CGCTGTTTTGACCTCCATAGAAGACACCGA	TGTTATCCTCCTCGCCCTTGCTCACACTAGTgctaccgcctcctccgct

FGR-siPcsk9	CGCTGTTTTGACCTCCATAGAAGACACCGA	TGTTATCCTCCTCGCCCTTGCTCACACTAGTgctaccgcctcctccgct
FYN-siPcsk9	CGCTGTTTTGACCTCCATAGAAGACACCGA	TGTTATCCTCCTCGCCCTTGCTCACACTAGTgctaccgcctcctccgct
RNF11-siPcsk9	CGCTGTTTTGACCTCCATAGAAGACACCGA	TGTTATCCTCCTCGCCCTTGCTCACACTAGTgctaccgcctcctccgct
Myr(FRS3)-siPcsk9	CGCTGTTTTGACCTCCATAGAAGACACCGA	TGTTATCCTCCTCGCCCTTGCTCACACTAGTgctaccgcctcctccgct
G2A-miR200a-3p	AGACACCGACTCTAGAggccgccaccATGgccAACCTGTTCGGCCGCAAGAAGCAGAG	ctaccaccgccgccACCGGTactAccgccGCGGCTCTGCTTCTTGCGGCCGAACA
G2A-siPcsk9	AGACACCGACTCTAGAggccgccaccATGgccAACCTGTTCGGCCGCAAGAAGCAGAG	ctaccaccgccgccACCGGTactAccgccGCGGCTCTGCTTCTTGCGGCCGAACA
Myr-TurboID	aggcggtagcACTAGTggcaagcccatccccaac	GTTGATTGTCGACTCAGGATCCctttcggcagaccgcagac
Primers for RT-qPCR		
GAPDH	TCGGAGTCAACGGATTTGGT	TTCCCGTTCTCAGCCTTGAC
U6	CTCGCTTCGGCAGCACA	AACGCTTCACGAATTTGCGT
miR-200a-3p	GCCCGCTAACACTGTCTGGTAA	CAGTGCGTGTCGTGGAGT
miR-125b-5p	GCTCCCTGAGACCCTAAC	CAGTGCGTGTCGTGGAGT
miR-451	GCGCAAACCGTTACCATTAC	CAGTGCGTGTCGTGGAGT
mRNA GFP	cgtgccatcatcctgcgc	catgcacttcaagagcgccatc
siRNA METTL3	CGCCCGTATCACAACAGATCC	CAGTGCGTGTCGTGGAGT
siRNA YAP	GCGCGCTTCTTTATCTAGCT	CAGTGCGTGTCGTGGAGT

siRNA EZH2	GCGCGCttttcctttagtt	CAGTGCGTGTCGTGGAGT
siRNA Pcsk9	GCGCGATGTTGATGACGT	CAGTGCGTGTCGTGGAGT
let-7i-5p	GCGCGTGAGGTAGTAGTTTGT	CAGTGCGTGTCGTGGAGT
ANXA2	GGTTGAACACATTGGCCTCAG	TGTTCAAAGCATCCCGCTCA
ALIX	CAAGTCTGTCAGCCAGTCAGT	AGCTGCACCGAGATGAATGT
TSG101	GTAGTGGTGCCGACTTCCTG	ACACCATTTTCTTGAGCTGGC
Primers for microRNA and siRNA reverse transcription		
Name	Primer (5’ -3’)	
miR-200a-3p-RT	GTCGTATCCAGTGCGTGTCGTGGAGTCGGCAATTGCACTGGATACGACACATCG	
miR-125b-5p-RT	GTCGTATCCAGTGCGTGTCGTGGAGTCGGCAATTGCACTGGATACGACTCACAA	
miR-451-RT	GTCGTATCCAGTGCGTGTCGTGGAGTCGGCAATTGCACTGGATACGACAACTCA	
METTL3 siRNA-RT	GTCGTATCCAGTGCGTGTCGTGGAGTCGGCAATTGCACTGGATACGACAACAGT	
EZH2-siRNA-RT	GTCGTATCCAGTGCGTGTCGTGGAGTCGGCAATTGCACTGGATACGACCAGAAG	
YAP1-siRNA-RT	GTCGTATCCAGTGCGTGTCGTGGAGTCGGCAATTGCACTGGATACGACCCACCA	
Pcsk9-siRNA#3-RT	GTCGTATCCAGTGCGTGTCGTGGAGTCGGCAATTGCACTGGATACGACCCAAAG	
let-7i-5p-RT	GTCGTATCCAGTGCGTGTCGTGGAGTCGGCAATTGCACTGGATACGACAACAGC	

517

Supplemental Table 3. Major Resources Table

Antibodies

Target antigen	Vendor or Source	Catalog #	Working concentration	Persistent ID / URL
KEAP1	Proteintech	10503-2-AP	WB: 1:2000	http://www.ptgcn.com/products/KEAP1-Antibody-10503-2-AP.htm
GAPDH	Proteintech	60004- 1-Ig	WB: 0. 1 pg/mL	https://www.ptgcn.com/products/GAPDH-Antibody-60004-1-Ig.htm
YAP1	Proteintech	13584-1-AP	WB: 1:2000	https://www.ptgcn.com/products/YAP1-Antibody-13584-1-AP.htm#tested-applications
EZH2	Proteintech	21800-1-AP	WB: 1:1000	https://www.ptgcn.com/products/EZH2-Antibody-21800-1-AP.htm#tested-applications
METTL3	CST	96391S	WB: 1:2000	https://www.cellsignal.cn/products/primary-antibodies/mettl3-d2i6o-rabbit-mab/96391
Calnexin	Proteintech	66903-1-Ig	WB: 1:10000	https://www.ptgcn.com/products/Calnexin-Antibody-66903-1-Ig.htm
CD81	Proteintech	66866-1-Ig	WB: 1:2000	https://www.ptgcn.com/products/CD81-Antibody-66866-1-Ig.htm

ALIX	Proteintech	12422-1-AP	WB: 1:2000	https://www.ptgcn.com/products/PDCD6IP-Antibody-12422-1-AP.htm
Pcsk9	Affinity	DF12687	WB: 1:2000	https://www.affbiotech.com/goods-15999-DF12687-PCSK9_Antibody.html
LDLR	Proteintech	10785-1-AP	WB: 1:2000<br>IF: 1:400	https://www.ptgcn.com/products/LDLR-Antibody-10785-1-AP.htm
p62	Proteintech	18420-1-AP	WB: 1:2000	https://www.ptgcn.com/products/SQSTM1-Antibody-18420-1-AP.htm
LC3	CST	12741S	WB: 1:1000	https://www.cellsignal.com/products/primary-antibodies/lc3a-b-d3u4c-rabbit-monoclonal-antibody/12741
p-AMPK	CST	2535S	WB: 1:1000	https://www.cellsignal.com/products/primary-antibodies/phospho-ampk-alpha-thr172-40h9-rabbit-monoclonal-antibody/2535
AMPK	CST	2532S	WB: 1:1000	https://www.cellsignal.com/products/primary-antibodies/ampk-alpha-antibody/2532
V5-tag	Proteintech	14440-1-AP	WB: 1:2000	https://www.ptgcn.com/products/V5-tag-Antibody-14440-1-AP.htm

ANXA2	Proteintech	11256-1-AP	WB: 1:5000	https://www.ptgcn.com/products/ANXA2-Antibody-11256-1-AP.htm
TSG101	CST	72312T	WB: 1:1000	https://www.cellsignal.cn/products/primary-antibodies/tsg101-e6v1x-rabbit-monoclonal-antibody/72312
α-Tubulin	Proteintech	14555-1-AP	WB: 1:5000	https://www.ptgcn.com/products/TUBA1A-Antibody-14555-1-AP.htm
p-ERK1/2	CST	4370T	WB：1：1000	https://www.cellsignal.com/products/primary-antibodies/phospho-p44-42-mapk-erk1-2-thr202-tyr204-d13-14-4e-rabbit-monoclonal-antibody/4370
ERK1/2	CST	4695T	WB：1：1000	https://www.cellsignal.com/products/primary-antibodies/p44-42-mapk-erk1-2-137f5-rabbit-monoclonal-antibody/4695
AGO2	CST	2897T	WB：1：1000	https://www.cellsignal.com/products/primary-antibodies/argonaute-2-c34c6-rabbit-monoclonal-antibody/2897

Cultured Cells

Name	Vendor or Source	Sex (F, M, or unknown)	Persistent ID / URL
HUVECs	ScienCell	unknown	https://www.sciencellonline.com/products-services/primary-cells/human/cell-types/endothelial-cells/human-umbilical-vein-endothelial-cells.html
Lenti-X 293T	Clontech	unknown	https://www.takarabio.com/products/gene-function/viral-transduction/lentivirus/packaging-systems-and-cells/lenti-x-293t-cells?catalog=632180
Hepa1-6	procell	unknown	https://www.procell.com.cn/view/8698.html

Vectors

Name	Vendor or Source	Catalog #
PsPAX2	addgene	Plasmid #12260
pMD2.G	addgene	Plasmid #12259

References

1. Fan J, Pan J, Zhang X, Chen Y, Zeng Y, Huang L, Ma D, Chen Z, Wu G, Fan W. A peptide derived from the N-terminus of charged multivesicular body protein 6 (CHMP6) promotes the secretion of gene editing proteins via small extracellular vesicle production. Bioengineered. 2022 Mar; 13 (3): 4702-4716. doi:10.1080/21655979.2022.2030571.
2. Cho KF, Branon TC, Udeshi ND, Myers SA, Carr SA, Ting AY. Proximity labeling in mammalian cells with TurboID and split-TurboID. Nat Protoc. 2020 Dec; 15 (12): 3971-3999. doi:10.1038/s41596-020-0399-0.
3. Yang D, Zhang W, Zhang H, Zhang F, Chen L, Ma L, Larcher LM, Chen S, Liu N, Zhao Q; et al. Progress, opportunity, and perspective on exosome isolation - efforts for efficient exosome-based theranostics. Theranostics. 2020 Feb 19; 10 (8): 3684-3707. doi:10.7150/thno.41580.
4. Lobb RJ, Becker M, Wen SW, Wong CS, Wiegmans AP, Leimgruber A, Moller A. Optimized exosome isolation protocol for cell culture supernatant and human plasma. J Extracell Vesicles. 2015 Jul 17; 4: 27031. doi:10.3402/jev.v4.27031.
